# Invariant visual object recognition: biologically plausible approaches

**DOI:** 10.1007/s00422-015-0658-2

**Published:** 2015-09-03

**Authors:** Leigh Robinson, Edmund T. Rolls

**Affiliations:** Department of Computer Science, University of Warwick, Coventry, UK; Oxford Centre for Computational Neuroscience, Oxford, UK

**Keywords:** Visual object recognition, Invariant representations, Inferior temporal visual cortex, VisNet, HMAX, Trace learning rule

## Abstract

Key properties of inferior temporal cortex neurons are described, and then, the biological plausibility of two leading approaches to invariant visual object recognition in the ventral visual system is assessed to investigate whether they account for these properties. Experiment 1 shows that VisNet performs object classification with random exemplars comparably to HMAX, except that the final layer C neurons of HMAX have a very non-sparse representation (unlike that in the brain) that provides little information in the single-neuron responses about the object class. Experiment 2 shows that VisNet forms invariant representations when trained with different views of each object, whereas HMAX performs poorly when assessed with a biologically plausible pattern association network, as HMAX has no mechanism to learn view invariance. Experiment 3 shows that VisNet neurons do not respond to scrambled images of faces, and thus encode shape information. HMAX neurons responded with similarly high rates to the unscrambled and scrambled faces, indicating that low-level features including texture may be relevant to HMAX performance. Experiment 4 shows that VisNet can learn to recognize objects even when the view provided by the object changes catastrophically as it transforms, whereas HMAX has no learning mechanism in its S–C hierarchy that provides for view-invariant learning. This highlights some requirements for the neurobiological mechanisms of high-level vision, and how some different approaches perform, in order to help understand the fundamental underlying principles of invariant visual object recognition in the ventral visual stream.

**Electronic supplementary material:**

The online version of this article (doi:10.1007/s00422-015-0658-2) contains supplementary material, which is available to authorized users.

## Introduction

The aim of this research is to assess the biological plausibility of two models that aim to be biologically plausible or at least biologically inspired by performing investigations of how biologically plausible they are and comparing them to the known responses of inferior temporal cortex neurons. Four key experiments are performed to measure the firing rate representations provided by neurons in the models: whether the neuronal representations are of individual objects or faces as well as classes; whether the neuronal representations are transform invariant; whether whole objects with the parts in the correct spatial configuration are represented; and whether the systems can correctly represent individual objects that undergo catastrophic view transforms. In all these cases, the performance of the models is compared to that of neurons in the inferior temporal visual cortex. The overall aim is to provide insight into what must be accounted for more generally by biologically plausible models of object recognition by the brain, and in this sense, the research described here goes beyond these two models. We do not consider non-biologically plausible models here as our aim is neuroscience, how the brain works, but we do consider in the Discussion some of the factors that make some other models not biologically plausible, in the context of guiding future investigations of biologically plausible models of how the brain solves invariant visual object recognition. We note that these biologically inspired models are intended to provide elucidation of some of the key properties of the cortical implementation of invariant visual object recognition, and of course as models the aim is to include some modelling simplifications, which are referred to below, in order to provide a useful and tractable model.

One of the major problems that are solved by the visual system in the primate including human cerebral cortex is the building of a representation of visual information that allows object and face recognition to occur relatively independently of size, contrast, spatial frequency, position on the retina, angle of view, lighting, etc. These invariant representations of objects, provided by the inferior temporal visual cortex (Rolls 2008, 2012a), are extremely important for the operation of many other systems in the brain, for if there is an invariant representation, it is possible to learn on a single trial about reward/punishment associations of the object, the place where that object is located, and whether the object has been seen recently, and then to correctly generalize to other views, etc., of the same object (Rolls 2008, 2014). In order to understand how the invariant representations are built, computational models provide a fundamental approach, for they allow hypotheses to be developed, explored and tested, and are essential for understanding how the cerebral cortex solves this major computation.

We next summarize some of the key and fundamental properties of the responses of primate inferior temporal cortex (IT) neurons (Rolls 2008, 2012a; Rolls and Treves 2011) that need to be addressed by biologically plausible models of invariant visual object recognition. Then we illustrate how models of invariant visual object recognition can be tested to reveal whether they account for these properties. The two leading approaches to visual object recognition by the cerebral cortex that are used to highlight whether these generic biological issues are addressed are VisNet (Rolls 2012a, 2008; Wallis and Rolls 1997; Rolls and Webb 2014; Webb and Rolls 2014) and HMAX (Serre et al. 2007c, a, b; Mutch and Lowe 2008). By comparing these models, and how they perform on invariant visual object recognition, we aim to make advances in the understanding of the cortical mechanisms underlying this key problem in the neuroscience of vision. The architecture and operation of these two classes of network are described below.

Some of the key properties of IT neurons that need to be addressed, and that are tested in this paper, include:

1. Inferior temporal visual cortex neurons show responses to objects that are typically translation, size, contrast, rotation, and in many cases view invariant, that is, they show transform invariance (Hasselmo et al. 1989; Tovee et al. 1994; Logothetis et al. 1995; Booth and Rolls 1998; Rolls 2012a; Trappenberg et al. 2002; Rolls and Baylis 1986; Rolls et al. 1985, 1987, 2003; Aggelopoulos and Rolls 2005).
2. Inferior temporal cortex neurons show sparse distributed representations, in which individual neurons have high firing rates to a few stimuli and lower firing rates to more stimuli, in which much information can be read from the responses of a single neuron from its firing rates (because they are high to relatively few stimuli), and in which neurons encode independent information about a set of stimuli, as least up to tens of neurons (Tovee et al. 1993; Rolls and Tovee 1995; Rolls et al. 1997a, b; Abbott et al. 1996; Baddeley et al. 1997; Rolls 2008, 2012a; Rolls and Treves 2011).
3. Inferior temporal cortex neurons often respond to objects and not to low-level features, in that many respond to whole objects, but not to the parts presented individually nor to the parts presented with a scrambled configuration (Perrett et al. 1982; Rolls et al. 1994).
4. Inferior temporal cortex neurons convey information about the individual object or face, not just about a class such as face versus non-face, or animal versus non-animal (Rolls and Tovee 1995; Rolls et al. 1997a, b; Abbott et al. 1996; Baddeley et al. 1997; Rolls 2008, 2012a; Rolls and Treves 2011). This key property is essential for recognizing a particular person or object and is frequently not addressed in models of invariant object recognition, which still focus on classification into, e.g. animal versus non-animal, hats versus bears versus beer mugs (Serre et al. 2007c, a, b; Mutch and Lowe 2008; Yamins et al. 2014).
5. The learning mechanism needs to be physiologically plausible, that is, likely to include a local synaptic learning rule (Rolls 2008). We note that lateral propagation of weights, as used in the neocognitron (Fukushima 1980), HMAX (Riesenhuber and Poggio 1999; Mutch and Lowe 2008; Serre et al. 2007a), and convolution nets (LeCun et al. 2010), is not biologically plausible.

**Fig. 1:**
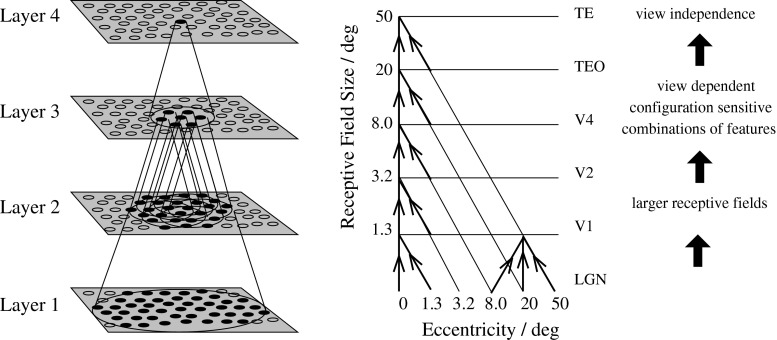
Convergence in the visual system. *Right* as it occurs in the brain. V1, visual cortex area V1; TEO, posterior inferior temporal cortex; TE, inferior temporal cortex (IT). *Left* as implemented in VisNet. Convergence through the network is designed to provide fourth layer neurons with information from across the entire input retina

## Methods

### Overview of the architecture of the ventral visual stream model, VisNet

The architecture of VisNet (Rolls 2008, 2012a) is summarized briefly next, with a full description provided after this.

Fundamental elements of Rolls’ (1992) theory for how cortical networks might implement invariant object recognition are described in detail elsewhere (Rolls 2008, 2012a). They provide the basis for the design of VisNet, which can be summarized as:

- A series of competitive networks organized in hierarchical layers, exhibiting mutual inhibition over a short range within each layer. These networks allow combinations of features or inputs occurring in a given spatial arrangement to be learned by neurons using competitive learning (Rolls 2008), ensuring that higher-order spatial properties of the input stimuli are represented in the network. In VisNet, layer 1 corresponds to V2, layer 2 to V4, layer 3 to posterior inferior temporal visual cortex, and layer 4 to anterior inferior temporal cortex. Layer one is preceded by a simulation of the Gabor-like receptive fields of V1 neurons produced by each image presented to VisNet (Rolls 2012a).
- A convergent series of connections from a localized population of neurons in the preceding layer to each neuron of the following layer, thus allowing the receptive field size of neurons to increase through the visual processing areas or layers, as illustrated in Fig. 1.
- A modified associative (Hebb-like) learning rule incorporating a temporal trace of each neuron’s previous activity, which, it has been shown (Földiák 1991; Rolls 1992, 2012a; Wallis et al. 1993; Wallis and Rolls 1997; Rolls and Milward 2000), enables the neurons to learn transform invariances.

The learning rates for each of the four layers were 0.05, 0.03, 0.005, and 0.005, as these rates were shown to produce convergence of the synaptic weights after 15–50 training epochs. Fifty training epochs were run.

### VisNet trace learning rule

The learning rule implemented in the VisNet simulations utilizes the spatio-temporal constraints placed upon the behaviour of ‘real-world’ objects to learn about natural object transformations. By presenting consistent sequences of transforming objects, the cells in the network can learn to respond to the same object through all of its naturally transformed states, as described by Földiák (1991), Rolls (1992, 2012a), Wallis et al. (1993), and Wallis and Rolls (1997). The learning rule incorporates a decaying trace of previous cell activity and is henceforth referred to simply as the ‘trace’ learning rule. The learning paradigm we describe here is intended in principle to enable learning of any of the transforms tolerated by inferior temporal cortex neurons, including position, size, view, lighting, and spatial frequency (Rolls 1992, 2000, 2008, 2012a; Rolls and Deco 2002).

Various biological bases for this temporal trace have been advanced as follows: The precise mechanisms involved may alter the precise form of the trace rule which should be used. Földiák (1992) describes an alternative trace rule which models individual NMDA channels. Equally, a trace implemented by temporally extended cell firing in a local cortical attractor could implement a short-term memory of previous neuronal firing (Rolls 2008).

- The persistent firing of neurons for as long as 100–400 ms observed after presentations of stimuli for 16 ms (Rolls and Tovee 1994) could provide a time window within which to associate subsequent images. Maintained activity may potentially be implemented by recurrent connections between as well as within cortical areas (Rolls and Treves 1998; Rolls and Deco 2002; Rolls 2008). The prolonged firing of anterior ventral temporal / perirhinal cortex neurons during memory delay periods of several seconds and associative links reported to develop between stimuli presented several seconds apart (Miyashita 1988) are on too long a time scale to be immediately relevant to the present theory. In fact, associations between visual events occurring several seconds apart would, under *normal* environmental conditions, be detrimental to the operation of a network of the type described here, because they would probably arise from different objects. In contrast, the system described benefits from associations between visual events which occur close in time (typically within 1 s), as they are likely to be from the same object.
- The binding period of glutamate in the NMDA channels, which may last for 100 ms or more, may implement a trace rule by producing a narrow time window over which the *average* activity at each presynaptic site affects learning (Rolls 1992; Rhodes 1992; Földiák 1992; Spruston et al. 1995; Hestrin et al. 1990).
- Chemicals such as nitric oxide may be released during high neural activity and gradually decay in concentration over a short time window during which learning could be enhanced (Földiák 1992; Montague et al. 1991; Garthwaite 2008).

The trace update rule used in the baseline simulations of VisNet (Wallis and Rolls 1997) is equivalent to both Földiák’s used in the context of translation invariance (Wallis et al. 1993) and the earlier rule of Sutton and Barto (1981) explored in the context of modelling the temporal properties of classical conditioning and can be summarized as follows:

1 $$\begin{aligned} \delta {w_j} = \alpha {\overline{y}}^{\tau } x_j \end{aligned}$$

where

2 $$\begin{aligned} {\overline{y}}^{\tau } = (1-\eta ){y}^{\tau }+{\eta \overline{y}}^{\tau -1} \end{aligned}$$

and $$ x_j$$: *j*th input to the neuron; $$ \overline{y}^{\tau }$$: Trace value of the output of the neuron at time step $$\tau $$; $$w_j$$: Synaptic weight between *j*th input and the neuron; *y*: Output from the neuron; $$\alpha $$: Learning rate; $$\eta $$: Trace value. The optimal value varies with presentation sequence length.

At the start of a series of investigations of different forms of the trace learning rule, Rolls and Milward (2000) demonstrated that VisNet’s performance could be greatly enhanced with a modified Hebbian trace learning rule (Eq. 3) that incorporated a trace of activity from the preceding time steps, with no contribution from the activity being produced by the stimulus at the current time step. This rule took the form

3 $$\begin{aligned} \delta {w_j} = \alpha {\overline{y}}^{\tau -1}x^{\tau }_j. \end{aligned}$$

The trace shown in Eq. 3 is in the postsynaptic term. The crucial difference from the earlier rule (see Eq. 1) was that the trace should be calculated up to only the preceding timestep. This has the effect of updating the weights based on the preceding activity of the neuron, which is likely given the spatio-temporal statistics of the visual world to be from previous transforms of the same object (Rolls and Milward 2000; Rolls and Stringer 2001). This is biologically not at all implausible, as considered in more detail elsewhere (Rolls 2008, 2012a), and this version of the trace rule was used in this investigation.

The optimal value of $\eta$ in the trace rule is likely to be different for different layers of VisNet. For early layers with small receptive fields, few successive transforms are likely to contain similar information within the receptive field, so the value for $\eta$ might be low to produce a short trace. In later layers of VisNet, successive transforms may be in the receptive field for longer, and invariance may be developing in earlier layers, so a longer trace may be beneficial. In practice, after exploration we used $\eta$ values of 0.6 for layer 2, and 0.8 for layers 3 and 4. In addition, it is important to form feature combinations with high spatial precision before invariance learning supported by a temporal trace starts, in order that the feature combinations and not the individual features have invariant representations (Rolls 2008, 2012a). For this reason, purely associative learning with no temporal trace was used in layer 1 of VisNet (Rolls and Milward 2000).

The following principled method was introduced to choose the value of the learning rate $\alpha$ for each layer. The mean weight change from all the neurons in that layer for each epoch of training was measured and was set so that with slow learning over 15–50 trials, the weight changes per epoch would gradually decrease and asymptote with that number of epochs, reflecting convergence. Slow learning rates are useful in competitive nets, for if the learning rates are too high, previous learning in the synaptic weights will be overwritten by large weight changes later within the same epoch produced if a neuron starts to respond to another stimulus (Rolls 2008). If the learning rates are too low, then no useful learning or convergence will occur. It was found that the following learning rates enabled good operation with the 100 transforms of each of 4 stimuli used in each epoch in the present investigation: Layer 1 $\alpha =0.05$; Layer 2 $\alpha =0.03$ (this is relatively high to allow for the sparse representations in layer 1); Layer 3 $\alpha =0.005$; Layer 4 $\alpha =0.005$.

To bound the growth of each neuron’s synaptic weight vector, $\mathbf {w}_i$ for the *i*th neuron, its length is explicitly normalized [a method similarly employed by Malsburg (1973) which is commonly used in competitive networks (Rolls 2008)]. An alternative, more biologically relevant implementation, using a local weight bounding operation which utilizes a form of heterosynaptic long-term depression (Rolls 2008), has in part been explored using a version of the Oja (1982) rule (see Wallis and Rolls 1997).

### Network implemented in VisNet

The network itself is designed as a series of hierarchical, convergent, competitive networks, in accordance with the hypotheses advanced above. The actual network consists of a series of four layers, constructed such that the convergence of information from the most disparate parts of the network’s input layer can potentially influence firing in a single neuron in the final layer—see Fig. 1. This corresponds to the scheme described by many researchers (Van Essen et al. 1992; Rolls 1992, 2008, for example) as present in the primate visual system—see Fig. 1. The forward connections to a cell in one layer are derived from a topologically related and confined region of the preceding layer. The choice of whether a connection between neurons in adjacent layers exists or not is based upon a Gaussian distribution of connection probabilities which roll-off radially from the focal point of connections for each neuron. (A minor extra constraint precludes the repeated connection of any pair of cells.) In particular, the forward connections to a cell in one layer come from a small region of the preceding layer defined by the radius in Table 1 which will contain approximately 67 % of the connections from the preceding layer. Table 1 shows the dimensions for the research described here, a (16$\times$) larger version than the version of VisNet used in most of our previous investigations, which utilized $32\times 32$ neurons per layer. For the research on view and translation invariance learning described here, we decreased the number of connections to layer 1 neurons to 100 (from 272), in order to increase the selectivity of the network between objects. We increased the number of connections to each neuron in layers 2–4 to 400 (from 100), because this helped layer 4 neurons to reflect evidence from neurons in previous layers about the large number of transforms (typically 100 transforms, from 4 views of each object and 25 locations) each of which corresponded to a particular object.

**Table 1:** VisNet dimensions

	Dimensions	No. of connections	Radius
Layer 4	$128\times 128$	400	48
Layer 3	$128\times 128$	400	36
Layer 2	$128\times 128$	400	24
Layer 1	$128\times 128$	100	24
Input layer	$256\times 256\times 16$	–	–

Figure 1 shows the general convergent network architecture used. Localization and limitation of connectivity in the network are intended to mimic cortical connectivity, partially because of the clear retention of retinal topology through regions of visual cortex. This architecture also encourages the gradual combination of features from layer to layer which has relevance to the binding problem, as described elsewhere (Rolls 2008, 2012a).

### Competition and lateral inhibition in VisNet

In order to act as a competitive network some form of mutual inhibition is required within each layer, which should help to ensure that all stimuli presented are evenly represented by the neurons in each layer. This is implemented in VisNet by a form of lateral inhibition. The idea behind the lateral inhibition, apart from this being a property of cortical architecture in the brain, was to prevent too many neurons that received inputs from a similar part of the preceding layer responding to the same activity patterns. The purpose of the lateral inhibition was to ensure that different receiving neurons coded for different inputs. This is important in reducing redundancy (Rolls 2008). The lateral inhibition is conceived as operating within a radius that was similar to that of the region within which a neuron received converging inputs from the preceding layer (because activity in one zone of topologically organized processing within a layer should not inhibit processing in another zone in the same layer, concerned perhaps with another part of the image). The lateral inhibition in this investigation used the parameters for $\sigma$ as shown in Table 3.

The lateral inhibition and contrast enhancement just described are actually implemented in VisNet2 (Rolls and Milward 2000) and VisNet (Perry et al. 2010) in two stages, to produce filtering of the type illustrated elsewhere (Rolls 2008, 2012a). The lateral inhibition was implemented by convolving the activation of the neurons in a layer with a spatial filter, *I*, where $\delta$ controls the contrast and $\sigma$ controls the width, and *a* and *b* index the distance away from the centre of the filter

4 $$\begin{aligned} I_{a,b} = {\left\{ \begin{array}{ll} -\delta e^{-\frac{a^2+b^2}{\sigma ^2}} &{} \text {if }a \ne 0\hbox { or }b \ne 0, \\ 1 - \sum \limits _{a \ne 0, b \ne 0} I_{a,b} &{} \text {if }a = 0\hbox { and } b = 0. \end{array}\right. } \end{aligned}$$

The second stage involves contrast enhancement. A sigmoid activation function was used in the way described previously (Rolls and Milward 2000):

5 $$\begin{aligned} y = \text {f}^{\text {sigmoid}}(r) = \frac{1}{1+\hbox {e}^{-2\beta (r-\alpha )}} \end{aligned}$$

where *r* is the activation (or firing rate) of the neuron after the lateral inhibition, *y* is the firing rate after the contrast enhancement produced by the activation function, and $\beta$ is the slope or gain and $\alpha$ is the threshold or bias of the activation function. The sigmoid bounds the firing rate between 0 and 1 so global normalization is not required. The slope and threshold are held constant within each layer. The slope is constant throughout training, whereas the threshold is used to control the sparseness of firing rates within each layer. The (population) sparseness of the firing within a layer is defined (Rolls and Treves 1998, 2011; Franco et al. 2007; Rolls 2008) as:

6 $$\begin{aligned} a = \frac{\left( \sum _i y_i/n\right) ^2}{\sum _i y_i^2/n} \end{aligned}$$

where *n* is the number of neurons in the layer. To set the sparseness to a given value, e.g. 5 %, the threshold is set to the value of the 95th percentile point of the activations within the layer.

The sigmoid activation function was used with parameters (selected after a number of optimization runs) as shown in Table 2.

**Table 2:** Sigmoid parameters

Layer	1	2	3	4
Percentile	99.2	98	88	95
Slope $\beta$	190	40	75	26

In addition, the lateral inhibition parameters are as shown in Table 3.

**Table 3:** Lateral inhibition parameters

Layer	1	2	3	4
Radius, $\sigma$	1.38	2.7	4.0	6.0
Contrast, $\delta$	1.5	1.5	1.6	1.4

### Input to VisNet

VisNet is provided with a set of input filters which can be applied to an image to produce inputs to the network which correspond to those provided by simple cells in visual cortical area 1 (V1). The purpose of this is to enable within VisNet the more complicated response properties of cells between V1 and the inferior temporal cortex (IT) to be investigated, using as inputs natural stimuli such as those that could be applied to the retina of the real visual system. This is to facilitate comparisons between the activity of neurons in VisNet and those in the real visual system, to the same stimuli. In VisNet no attempt is made to train the response properties of simple cells, but instead we start with a defined series of filters to perform fixed feature extraction to a level equivalent to that of simple cells in V1, as have other researchers in the field (Hummel and Biederman 1992; Buhmann et al. 1991; Fukushima 1980), because we wish to simulate the more complicated response properties of cells between V1 and the inferior temporal cortex (IT). The elongated orientation-tuned input filters used in accord with the general tuning profiles of simple cells in V1 (Hawken and Parker 1987) and were computed by Gabor filters. Each individual filter is tuned to spatial frequency (0.0626–0.5 cycles/pixel over four octaves); orientation ($0^{\circ }$ to $135^{\circ }$ in steps of $45^{\circ }$); and sign ($\pm 1$). Of the 100 layer 1 connections, the number to each group in VisNet is shown in Table 4. Any zero D.C. filter can of course produce a negative as well as positive output, which would mean that this simulation of a simple cell would permit negative as well as positive firing. The response of each filter is zero thresholded and the negative results used to form a separate anti-phase input to the network. The filter outputs are also normalized across scales to compensate for the low-frequency bias in the images of natural objects.

**Table 4:** VisNet layer 1 connectivity

Frequency	0.5	0.25	0.125	0.0625
No. of connections	74	19	5	2

The frequency is in cycles per pixel

**Fig. 2:**
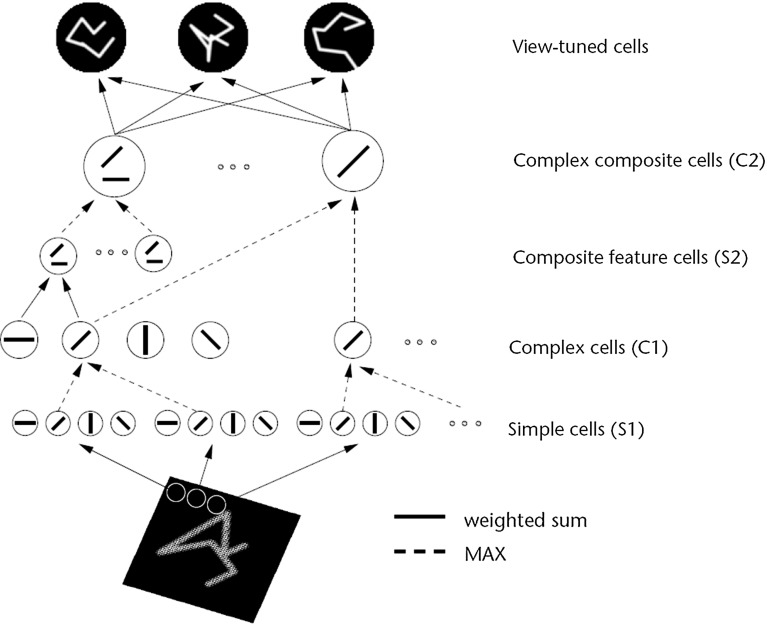
Sketch of Riesenhuber and Poggio (1999) HMAX model of invariant object recognition. The model includes layers of ‘S’ cells, which perform template matching (*solid lines*), and ‘C’ cells (*solid lines*), which pool information by a non-linear MAX function to achieve invariance (see text) (After Riesenhuber and Poggio 1999.)

The Gabor filters used were similar to those used previously (Deco and Rolls 2004; Rolls 2012a; Rolls and Webb 2014; Webb and Rolls 2014). Following Daugman (1988) the receptive fields of the simple cell-like input neurons are modelled by 2D Gabor functions. The Gabor receptive fields have five degrees of freedom given essentially by the product of an elliptical Gaussian and a complex plane wave. The first two degrees of freedom are the 2D locations of the receptive field’s centre; the third is the size of the receptive field; the fourth is the orientation of the boundaries separating excitatory and inhibitory regions; and the fifth is the symmetry. This fifth degree of freedom is given in the standard Gabor transform by the real and imaginary part, i.e. by the phase of the complex function representing it, whereas in a biological context this can be done by combining pairs of neurons with even and odd receptive fields. This design is supported by the experimental work of Pollen and Ronner (1981), who found simple cells in quadrature-phase pairs. Even more, Daugman (1988) proposed that an ensemble of simple cells is best modelled as a family of 2D Gabor wavelets sampling the frequency domain in a log-polar manner as a function of eccentricity. Experimental neurophysiological evidence constrains the relation between the free parameters that define a 2D Gabor receptive field (De Valois and De Valois 1988). There are three constraints fixing the relation between the width, height, orientation, and spatial frequency (Lee 1996). The first constraint posits that the aspect ratio of the elliptical Gaussian envelope is 2:1. The second constraint postulates that the plane wave tends to have its propagating direction along the short axis of the elliptical Gaussian. The third constraint assumes that the half-amplitude bandwidth of the frequency response is about 1–1.5 octaves along the optimal orientation. Further, we assume that the mean is zero in order to have an admissible wavelet basis (Lee 1996). Cells of layer 1 receive a topologically consistent, localized, random selection of the filter responses in the input layer, under the constraint that each cell samples every filter spatial frequency and receives a constant number of inputs. The mathematical details of the Gabor filtering are described elsewhere (Rolls 2012a; Rolls and Webb 2014; Webb and Rolls 2014).

### Recent developments in VisNet implemented in the research described here

The version of VisNet used in this paper differed from the versions used for most of the research published with VisNet before 2012 (Rolls 2012a) in the following ways. First, Gabor filtering was used here, with a full mathematical description provided here, as compared to the difference of Gaussian filters used earlier. Second, the size of VisNet was increased from the previous $32\times 32$ neurons per layer to the $128\times 128$ neurons per layer described here. Third, the steps described in the Method to set the learning rates $\alpha$ to values for each layer that encouraged convergence in 20–50 learning epochs were utilized here. Fourth, the method of pattern association decoding described in Sect. 2.8.2 to provide a biologically plausible way of decoding the outputs of VisNet neurons was used in the research described here. Fuller descriptions of the rationale for the design of VisNet, and of alternative more powerful learning rules not used here, are provided elsewhere (Rolls 2008, 2012a; Rolls and Stringer 2001).

### HMAX models used for comparison with VisNet

The performance of VisNet was compared against a standard HMAX model (Mutch and Lowe 2008; Serre et al. 2007a, b). We note that an HMAX family model has in the order of 10 million computational units (Serre et al. 2007a), which is at least 100 times the number contained within the current implementation of VisNet (which uses $128\times 128$ neurons in each of 4 layers, i.e. 65,536 neurons). HMAX has as an ancestor the neocognitron (Fukushima 1980, 1988), which is also a hierarchical network that uses lateral copying of filter analysers within each layer. Both approaches select filter analysers using feedforward processing without a teacher, in contrast to convolutional and deep learning networks (LeCun et al. 2010) which typically use errors from a teacher backpropagated through multiple layers that do not aim for biological plausibility (Rolls 2008, 2016).

HMAX is a multiple layer system with simple and complex cell layers alternating that sets up connections to simple cells based on randomly chosen exemplars, and a MAX function performed by the complex cells of their simple cell inputs. The inspiration for this architecture Riesenhuber and Poggio (1999) may have come from the simple and complex cells found in V1 by Hubel and Wiesel (1968). A diagram of the model as described by Riesenhuber and Poggio (1999) is shown in Fig. 2. The final complex cell layer is then typically used as an input to a non-biologically plausible support vector machine or least squares computation to perform classification of the representations into object classes. The inputs to both HMAX and VisNet are Gabor-filtered images intended to approximate V1. One difference is that VisNet is normally trained on images generated by objects as they transform in the world, so that view, translation, size, rotation, etc., invariant representations of objects can be learned by the network. In contrast, HMAX is typically trained with large databases of pictures of different exemplars of, for example, hats and beer mugs as in the Caltech databases, which do not provide the basis for invariant representations of objects to be learned, but are aimed at object classification.

When assessing the biological plausibility of the output representations of HMAX, we used the implementation of the HMAX model described by Mutch and Lowe (2008) using the code available at http://cbcl.mit.edu/jmutch/cns/index.html#hmax. In this instantiation of HMAX with 2 layers of S–C units, the assessment of performance was typically made using a support vector machine applied to the top layer C neurons. However, that way of measuring performance is not biologically plausible. However, Serre et al. (2007a) took the C2 neurons as corresponding to V4 and following earlier work in which view-tuned units were implemented (Riesenhuber and Poggio 1999) added a set of view-tuned units (VTU) which might be termed an S3 layer which they suggest corresponds to the posterior inferior temporal visual cortex. We implemented these VTUs in the way described by Riesenhuber and Poggio (1999) and Serre et al. (2007a) with an S3 VTU layer, by setting up a moderate number of view-tuned units, each one of which is set to have connection weights to all neurons in the C2 layer that reflect the firing rate of each C2 unit to one exemplar of a class. (This will produce the firing for any VTU that would be produced by one of the training views or exemplars of a class.) The S3 units that we implemented can thus be thought of as representing posterior inferior temporal cortex neurons (Serre et al. 2007a). The VTU output is classified by a one-layer error minimization network, i.e. a perceptron with one neuron for each class.

To ensure that the particular implementation of HMAX that we used for the experiments described in the main text, that of Mutch and Lowe (2008), was not different generically in the results obtained from other implementations of HMAX, we performed further investigations with the version of HMAX described by Serre et al. (2007a), which has 3 S–C layers. The S3 layer is supposed to correspond to posterior inferior temporal visual cortex, and the C3 layer, which is followed by S4 view-tuned units, to anterior inferior temporal visual cortex. The results with this version of HMAX were found to be generically similar in our investigations to those with the version implemented by Mutch and Lowe (2008), and the results with the version described by Serre et al. (2007a) are described in the Supplementary Material. We note that for both these versions of HMAX, the code is available at http://cbcl.mit.edu/jmutch/cns/index.html#hmax and that code defines the details of the architecture and the parameters, which were used unless otherwise stated, and for that reason the details of the HMAX implementations are not considered in great detail here. In the Supplementary Material, we do provide some further information about the HMAX version implemented by Serre et al. (2007a) which we used for the additional investigations reported in the Supplementary Material.

### Measures for network performance

#### Information theory measures

The performance of VisNet was measured by Shannon information-theoretic measures that are identical to those used to quantify the specificity and selectiveness of the representations provided by neurons in the brain (Rolls and Milward 2000; Rolls 2012a; Rolls and Treves 2011). A single cell information measure indicated how much information was conveyed by the firing rates of a single neuron about the most effective stimulus. A multiple cell information measure indicated how much information about every stimulus was conveyed by the firing rates of small populations of neurons and was used to ensure that all stimuli had some neurons conveying information about them.

A neuron can be said to have learnt an invariant representation if it discriminates one set of stimuli from another set, across all transforms. For example, a neuron’s response is translation invariant if its response to one set of stimuli irrespective of presentation is consistently higher than for all other stimuli irrespective of presentation location. Note that we state ‘set of stimuli’ since neurons in the inferior temporal cortex are not generally selective for a single stimulus but rather a subpopulation of stimuli (Baylis et al. 1985; Abbott et al. 1996; Rolls et al. 1997a; Rolls and Treves 1998, 2011; Rolls and Deco 2002; Rolls 2007; Franco et al. 2007; Rolls 2008). We used measures of network performance (Rolls and Milward 2000) based on information theory and similar to those used in the analysis of the firing of real neurons in the brain (Rolls 2008; Rolls and Treves 2011). A single cell information measure was introduced which is the maximum amount of information the cell has about any one object independently of which transform (here position on the retina and view) is shown. Because the competitive algorithm used in VisNet tends to produce local representations (in which single cells become tuned to one stimulus or object), this information measure can approach $\log _2 N_S$ bits, where $N_S$ is the number of different stimuli. Indeed, it is an advantage of this measure that it has a defined maximal value, which enables how well the network is performing to be quantified. Rolls and Milward (2000) also introduced a multiple cell information measure used here, which has the advantage that it provides a measure of whether all stimuli are encoded by different neurons in the network. Again, a high value of this measure indicates good performance.

For completeness, we provide further specification of the two information-theoretic measures, which are described in detail by Rolls and Milward (2000) (see Rolls 2008 and Rolls and Treves 2011 for an introduction to the concepts). The measures assess the extent to which either a single cell or a population of cells responds to the same stimulus invariantly with respect to its location, yet responds differently to different stimuli. The measures effectively show what one learns about which stimulus was presented from a single presentation of the stimulus at any randomly chosen transform. Results for top (4th) layer cells are shown. High information measures thus show that cells fire similarly to the different transforms of a given stimulus (object) and differently to the other stimuli. The single cell stimulus-specific information, *I*(*s*, *R*), is the amount of information the set of responses, *R*, has about a specific stimulus, *s* (see Rolls et al. 1997b and Rolls and Milward 2000). *I*(*s*, *R*) is given by

7 $$\begin{aligned} I(s,R) = \sum _{r \in R} P(r|s) \log _2 \frac{P(r|s)}{P(r)} \end{aligned}$$

where *r* is an individual response from the set of responses *R* of the neuron. For each cell the performance measure used was the maximum amount of information a cell conveyed about any one stimulus. This (rather than the mutual information, *I*(*S*, *R*) where *S* is the whole set of stimuli *s*) is appropriate for a competitive network in which the cells tend to become tuned to one stimulus. (*I*(*s*, *R*) has more recently been called the stimulus-specific surprise (DeWeese and Meister 1999; Rolls and Treves 2011). Its average across stimuli is the mutual information *I*(*S*, *R*).)

If all the output cells of VisNet learned to respond to the same stimulus, then the information about the set of stimuli *S* would be very poor and would not reach its maximal value of $\log _2$ of the number of stimuli (in bits). The second measure that is used here is the information provided by a set of cells about the stimulus set, using the procedures described by Rolls et al. (1997a) and Rolls and Milward (2000). The multiple cell information is the mutual information between the whole set of stimuli *S* and of responses *R* calculated using a decoding procedure in which the stimulus $s'$ that gave rise to the particular firing rate response vector on each trial is estimated. (The decoding step is needed because the high dimensionality of the response space would lead to an inaccurate estimate of the information if the responses were used directly, as described by Rolls et al. 1997a and Rolls and Treves 1998.) A probability table is then constructed of the real stimuli *s* and the decoded stimuli $s'$. From this probability table, the mutual information between the set of actual stimuli *S* and the decoded estimates $S'$ is calculated as

8 $$\begin{aligned} I(S, S') = \sum _{s,s'} P(s,s') \log _2 \frac{P(s,s')}{P(s)P(s')} \end{aligned}$$

This was calculated for the subset of cells which had as single cells the most information about which stimulus was shown. In particular, in Rolls and Milward (2000) and subsequent papers, the multiple cell information was calculated from the first five cells for each stimulus that had maximal single cell information about that stimulus, that is, from a population of 35 cells if there were seven stimuli (each of which might have been shown in, for example, 9 or 25 positions on the retina).

#### Pattern association decoding

In addition, the performance was measured by a biologically plausible one-layer pattern association network using an associative synaptic modification rule. There was one output neuron for each class (which was set to a firing rate of 1.0 during training of that class but was otherwise 0.0) and 10 input neurons per class to the pattern associator. These 10 neurons for each class were the most selective neurons in the output layer of VisNet or HMAX to each object. The most selective output neurons of VisNet and HMAX were identified as those with the highest mean firing rate to all transforms of an object relative to the firing rates across all transforms of all objects and a high corresponding stimulus-specific information value for that class. Performance was measured as the per cent correct object classification measured across all views of all objects.

The output of the inferior temporal visual cortex reaches structures such as the orbitofrontal cortex and amygdala, where associations to other stimuli are learned by a pattern association network with an associative (Hebbian) learning rule (Rolls 2008, 2014). We therefore used a one-layer pattern association network (Rolls 2008) to measure how well the output of VisNet could be classified into one of the objects. The pattern association network had one output neuron for each object or class. The inputs were the 10 neurons from layer 4 of VisNet for each of the objects with the best single cell information and high firing rates. For HMAX, the inputs were the 10 neurons from the C2 layer (or from 5 of the view-tuned units) for each of the objects with the highest mean firing rate for the class when compared to the firing rates over all the classes. The network was trained with the Hebb rule:

9 $$\begin{aligned} \delta {w_{ij}} = \alpha y_i x_j \end{aligned}$$

where $\delta {w_{ij}}$ is the change of the synaptic weight $w_{ij}$ that results from the simultaneous (or conjunctive) presence of presynaptic firing $x_j$ and postsynaptic firing or activation $y_i$, and $\alpha$ is a learning rate constant that specifies how much the synapses alter on any one pairing. The pattern associator was trained for one trial on the output of VisNet produced by every transform of each object.

Performance on the training or test images was tested by presenting an image to VisNet and then measuring the classification produced by the pattern associator. Performance was measured by the percentage of the correct classifications of an image as the correct object.

This approach to measuring the performance is very biologically appropriate, for it models the type of learning thought to be implemented in structures that receive information from the inferior temporal visual cortex such as the orbitofrontal cortex and amygdala (Rolls 2008, 2014). The small number of neurons selected from layer 4 of VisNet might correspond to the most selective for this stimulus set in a sparse distributed representation (Rolls 2008; Rolls and Treves 2011). The method would measure whether neurons of the type recorded in the inferior temporal visual cortex with good view and position invariance are developed in VisNet. In fact, an appropriate neuron for an input to such a decoding mechanism might have high firing rates to all or most of the view and position transforms of one of the stimuli, and smaller or no responses to any of the transforms of other objects, as found in the inferior temporal cortex for some neurons (Hasselmo et al. 1989; Perrett et al. 1991; Booth and Rolls 1998), and as found for VisNet layer 4 neurons (Rolls and Webb 2014). Moreover, it would be inappropriate to train a device such as a support vector machine or even an error correction perceptron on the outputs of all the neurons in layer 4 of VisNet to produce 4 classifications, for such learning procedures, not biologically plausible (Rolls 2008), could map the responses produced by a multilayer network with untrained random weights to obtain good classifications.

## Results

### Categorization of objects from benchmark object image sets: Experiment 1

The performance of HMAX and VisNet was compared on a test that has been used to measure the performance of HMAX (Mutch and Lowe 2008; Serre et al. 2007a, b) and indeed typical of many approaches in computer vision, the use of standard datasets such as the CalTech-256 (Griffin et al. 2007) in which sets of images from different object classes are to be classified into the correct object class.

#### Object benchmark database

**Fig. 3:**
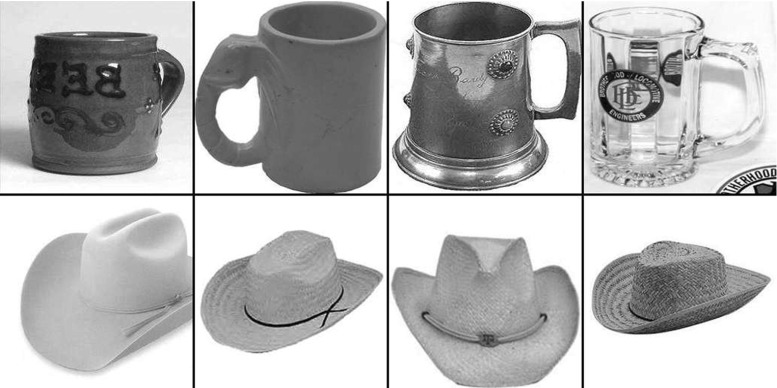
Example images from the Caltech256 database for two object classes, hats and beer mugs

The Caltech-256 dataset (Griffin et al. 2007) is comprised of 256 object classes made up of images that have many aspect ratios and sizes and differ quite significantly in quality (having being manually collated from web searches). The objects within the images show significant intra-class variation and have a variety of poses, illumination, scale, and occlusion as expected from natural images (see examples in Fig. 3). In this sense, the Caltech-256 database has been considered to be a difficult challenge to object recognition systems. We come to the conclusion below that the benchmarking approach with this type of dataset is not useful for training a system that must learn invariant object representations. The reason for this is that the exemplars of each object class in the CalTech-256 dataset are too discontinuous to provide a basis for learning transform-invariant object representations. For example, the image exemplars within an object class in these datasets may be very different indeed.

#### Performance on a Caltech-256 test

**Fig. 4:**
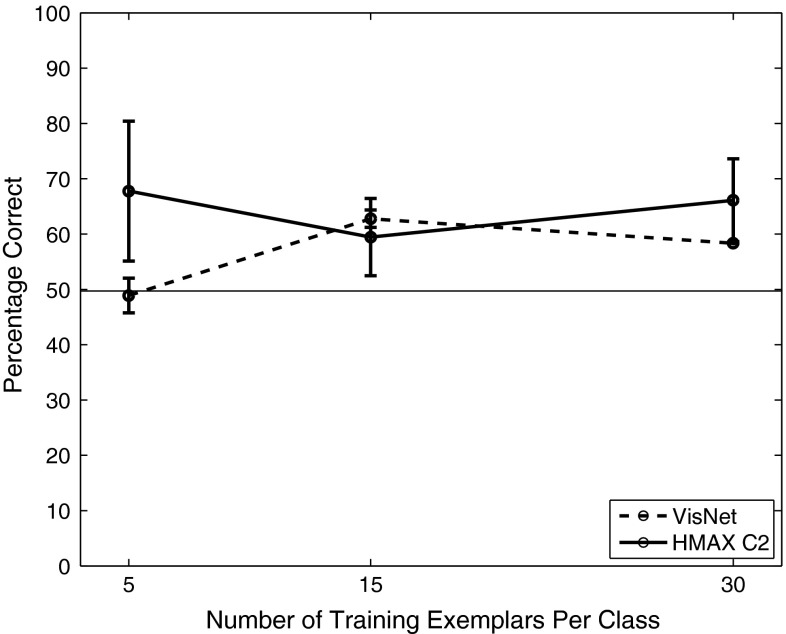
Performance of HMAX and VisNet on the classification task (measured by the proportion of images classified correctly) using the Caltech-256 dataset and linear support vector machine (SVM) classification. The *error bars* show the standard deviation of the means over three cross-validation trials with different images chosen at random for the training set on each trial. There were two object classes, hats and beer mugs, with the number of training exemplars shown on the abscissa. There were 30 test examples of each object class. All cells in the C2 layer of HMAX and layer 4 of Visnet were used to measure the performance. Chance performance at 50 % is indicated

VisNet and the HMAX model were trained to discriminate between two object classes from the Caltech-256 database, the *beer mugs* and *cowboy-hat* (see examples in Fig. 3). The images in each class were rescaled to $256\times 256$ and converted to grayscale, so that shape recognition was being investigated. The images from each class were randomly partitioned into training and testing sets with performance measured in this cross-validation design over multiple random partitions. Figure 4 shows the performance of the VisNet and HMAX models when performing the task with these exemplars of the Caltech-256 dataset. Performance of HMAX and VisNet on the classification task was measured by the proportion of images classified correctly using a linear support vector machine (SVM) on all the C2 cells in HMAX [chosen as the way often used to test the performance of HMAX (Mutch and Lowe 2008; Serre et al. 2007a, b)] and on all the layer 4 (output layer) cells of VisNet. The error bars show the standard deviation of the means over three cross-validation trials with different images chosen at random for the training set and test set on each trial. The number of training exemplars is shown on the abscissa. There were 30 test examples of each object class. Chance performance at 50 % is indicated. Performance of HMAX and VisNet was similar, but was poor, probably reflecting the fact that there is considerable variation of the images within each object class, making the cross-validation test quite difficult. The nature of the performance of HMAX and VisNet on this task is assessed in the next section.

#### Biological plausibility of the neuronal representations of objects that are produced

In the temporal lobe visual cortical areas, neurons represent which object is present using a sparse distributed representation (Rolls and Treves 2011). Neurons typically have spontaneous firing rates of a few spikes/s and increase their firing rates to 30–100 spikes/s for effective stimuli. Each neuron responds with a graded range of firing rates to a small proportion of the stimuli in what is therefore a sparse representation (Rolls and Tovee 1995; Rolls et al. 1997b). The information can be read from the firing of single neurons about which stimulus was shown, with often 2–3 bits of stimulus-specific information about the most effective stimulus (Rolls et al. 1997b; Tovee et al. 1993). The information from different neurons increases approximately linearly with the number of neurons recorded (up to approximately 20 neurons), indicating independent encoding by different neurons (Rolls et al. 1997a). The information from such groups of responsive neurons can be easily decoded (using, for example, dot product decoding utilizing the vector of firing rates of the neurons) by a pattern association network (Rolls et al. 1997a; Rolls 2008, 2012a; Rolls and Treves 2011). This is very important for biological plausibility, for the next stage of processing, in brain regions such as the orbitofrontal cortex and amygdala, contains pattern association networks that associate the outputs of the temporal cortex visual areas with stimuli such as taste (Rolls 2008, 2014).

We therefore compared VisNet and HMAX in the representations that they produce of objects, to analyse whether they produce these types of representation, which are needed for biological plausibility. We note that the usual form of testing for VisNet does involve the identical measures used to measure the information present in the firing of temporal cortex neurons with visual responses (Rolls and Milward 2000; Rolls 2012a; Rolls et al. 1997a, b). On the other hand, the output of HMAX is typically read and classified by a powerful and artificial support vector machine (Mutch and Lowe 2008; Serre et al. 2007a, b), so it is necessary to test its output with the same type of biologically plausible neuronal firing rate decoding used by VisNet. Indeed, the results shown in Sect. 3.1.2 were obtained with support vector machine decoding used for both HMAX and VisNet. In this section, we analyse the firing rate representations produced by VisNet and HMAX, to assess the biological plausibility of their output representations. The information measurement procedures are described in Sect. 2.8, and in more detail elsewhere (Rolls and Milward 2000; Rolls 2012a; Rolls et al. 1997a, b).

**Fig. 5:**
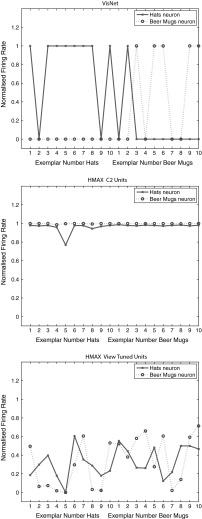
*Top* firing rate of two output layer neurons of VisNet, when tested on two of the classes, hats and beer mugs, from the Caltech 256. The firing rates to 10 untrained (i.e. testing) exemplars of each of the two classes are shown. One of the neurons responded more to hats than to beer mugs (*solid line*). The other neuron responded more to beer mugs than to hats (*dashed line*). *Middle* firing rate of two C2 tuned units of HMAX when tested on two of the classes, beer mugs and hats, from the Caltech 256. *Bottom* firing rate of a view-tuned unit of HMAX when tested on two of the classes, hats (*solid line*) and beer mugs (*dashed line*), from the Caltech 256. The neurons chosen were those with the highest single cell information that could be decoded from the responses of a neuron to 10 exemplars of each of the two objects (as well as a high firing rate) in the cross-validation design

Figure 5 Upper shows the firing rates of two VisNet neurons for the test set, in the experiment with the Caltech-256 dataset using two object classes, beer mugs and hats, when trained on 50 exemplars of each class, and then tested in a cross-validation design with 10 test exemplars of each class that had not been seen during training.

For the testing (untrained, cross validation) set of exemplars, one of the neurons responded with a high rate to 8 of the 10 untrained exemplars of one class (hats) and to 1 of the exemplars of the other class (beer mugs). The single cell information was 0.38 bits. The other neuron responded to 5 exemplars of the beer mugs class and to no exemplars of the hats class, and its single cell information was 0.21 bits. The mean stimulus-specific single cell information across the 5 most informative cells for each class was 0.28 bits.

The results for the cross-validation testing mode shown in Fig. 5(upper) thus show that VisNet can learn about object classes and can perform reasonable classification of untrained exemplars. Moreover, these results show that VisNet can do this using simple firing rate encoding of its outputs, which might potentially be decoded by a pattern associator. To test this, we trained a pattern association network on the output of VisNet to compare with the support vector machine results shown in Fig. 4. With 30 training exemplars, classification using the best 10 neurons for each class was 61.7 % correct, compared to chance performance of 50 % correct.

Figure 5 (middle) shows two neurons in the C2 and (bottom) two neurons in the view-tuned unit layer of HMAX on the test set of 10 exemplars of each class in the same task. It is clear that the C2 neurons both responded to all 10 untrained exemplars of both classes, with high firing rates to almost presented images. The normalized mean firing rate of one of the neurons was 0.905 to the beer mugs and 0.900 to the hats. We again used a pattern association network on the output of HMAX C2 neurons to compare with the support vector machine results shown in Fig. 4. With 30 training exemplars, classification using the best 10 neurons for each class was 63 % correct, compared to chance performance of 50 % correct. When biologically plausible decoding by an associative pattern association network is used, the performance of HMAX is poorer than when the performance of HMAX is measured with powerful least squares classification. The mean stimulus-specific single cell information across the 5 most informative cells for each class was 0.07 bits. This emphasizes that the output of HMAX is not in a biologically plausible form.

The relatively poor performance of VisNet (which produces a biologically plausible output), and of HMAX when its performance is measured in a biologically plausible way, raises the point that training with a diverse sets of exemplars of an object class as in the Caltech dataset is not a very useful way to test object recognition networks of the type found in the brain. Instead, the brain produces view-invariant representations of objects, using information about view invariance simply not present in the Caltech type of dataset, because it does not provide training exemplars shown with different systematic transforms (position over up to 70$^{\circ }$, size, rotation and view) for transform invariance learning. In the next experiment, we therefore investigated the performance of HMAX and VisNet with a dataset in which different views of each object class are provided, to compare how HMAX and VisNet perform on this type of problem.

**Fig. 6:**
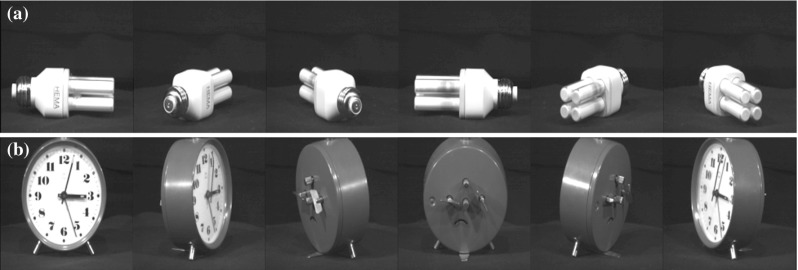
Example images from the two object classes within the ALOI database, **a** 293 (light bulb) and **b** 156 (clock). Only the $45^{\circ }$ increments are shown

Figure 5 (bottom) shows the firing rates of two view-tuned layer units of HMAX. It is clear that the view-tuned neurons had lower firing rates (and this is just a simple function of the value chosen for $\sigma$, which in this case was 1), but that again the firing rates differed little between the classes. For example, the mean firing rate of one of the VTU neurons to the beer mugs was 0.3 and to the hats was 0.35. The single cell stimulus-specific information measures were 0.28 bits for the hats neuron and 0.24 bits for the beer mugs neuron. The mean stimulus-specific single cell information across the 5 most informative VTUs for each class was 0.10 bits.

We note that if the VTU layer was classified with a least squares classifier (i.e. a perceptron, which is not biologically plausible, but is how the VTU neurons were decoded by Serre et al. 2007a), then performance was at 67 %. (With a pattern associator, the performance was 66 % correct.) Thus the performance of the VTU outputs (introduced to make the HMAX outputs otherwise of C neuron appear more biologically plausible) was poor on this type of CalTech-256 problem when measured both by a linear classifier and by a pattern association network.

Figure 1 of the Supplementary Material shows that similar results were obtained for the HMAX implementation by Serre et al. (2007a).

#### Evaluation of categorization when tested with large numbers of images presented randomly

The benchmark type of test using large numbers of images of different object classes presented in random sequence has limitations, in that an object can look quite different from different views. Catastrophic changes in the image properties of objects can occur as they are rotated through different views (Koenderink 1990). One example is that any view from above a cup into the cup that does not show the sides of the cup may look completely different from any view where some of the sides or bottom of the cup are shown. In this situation, training any network with images presented in a random sequence (i.e. without a classification label for each image) is doomed to failure in view-invariant object recognition. This applies to all such approaches that are unsupervised and that attempt to categorize images into objects based on image statistics. If a label for its object category is used for each image during training, this may help to produce good classification, but is very subject to over-fitting, in which small pixel changes in an image that do not affect which object it is interpreted as by humans may lead to it being misclassified (Krizhevsky et al. 2012; Szegedy et al. 2014).

In contrast, the training of VisNet is based on the concept that the transforms of an object viewed from different angles in the natural world provide the information required about the different views of an object to build a view-invariant representation and that this information can be linked together by the continuity of this process in time. Temporal continuity (Rolls 2012a) or even spatial continuity (Stringer et al. 2006; Perry et al. 2010) and typically both (Perry et al. 2006) provide the information that enables different images of an object to be associated together. Thus two factors, continuity of the image transforms as the object transforms through different views, and a principle of spatio-temporal closeness to provide a label of the object based on its property of spatio-temporal continuity, provide a principled way for VisNet, and it is proposed for the real visual system of primates including humans, to build invariant representations of objects (Rolls 1992, 2008, 2012a). This led to Experiment 2.

### Performance with the Amsterdam library of images: Experiment 2

Partly because of the limitations of the Caltech-256 database for training in invariant object recognition, we also investigated training with the Amsterdam Library of Images (ALOI) database (Geusebroek et al. 2005) (http://staff.science.uva.nl/~aloi/). The ALOI database takes a different approach to the Caltech-256 and instead of focussing on a set of natural images within an object category or class, provides images of objects with a systematic variation of pose and illumination for 1000 small objects. Each object is placed onto a turntable and photographed in consistent conditions at $5^{\circ }$ increments, resulting in a set of images that not only show the whole object (with regard to out of plane rotations), but does so with some continuity from one image to the next (see examples in Fig. 6).

Eight classes of object (with designations *156, 203, 234, 293, 299, 364, 674, 688*) from the dataset were chosen (see Fig. 6 for examples). Each class or object comprises of 72 images taken at $5^{\circ }$ increments through the full $360^{\circ }$ horizontal plane of rotation. Three sets of training images were used as follows. The training set consisted of 4 views of each object spaced $90^{\circ }$ apart; 9 views spaced $40^{\circ }$ apart; or 18 views spaced $20^{\circ }$ apart. The test set of images was in all cases a cross-validation set of 18 views of each object spaced $20^{\circ }$ apart and offset by $10^{\circ }$ from the training set with 18 views and not including any training view. The aim of using the different training sets was to investigate how close in viewing angle the training images need to be and also to investigate the effects of using different numbers of training images. The performance was measured with a pattern association network with one neuron per object and 10 inputs for each class that were the most selective neurons for an object in the output layer of VisNet or the C2 layer of HMAX. The best cells of VisNet or HMAX for a class were selected as those with the highest mean rate across views to the members of that class relative to the firing rate to all views of all objects and with a high stimulus-specific information for that class.

**Fig. 7:**
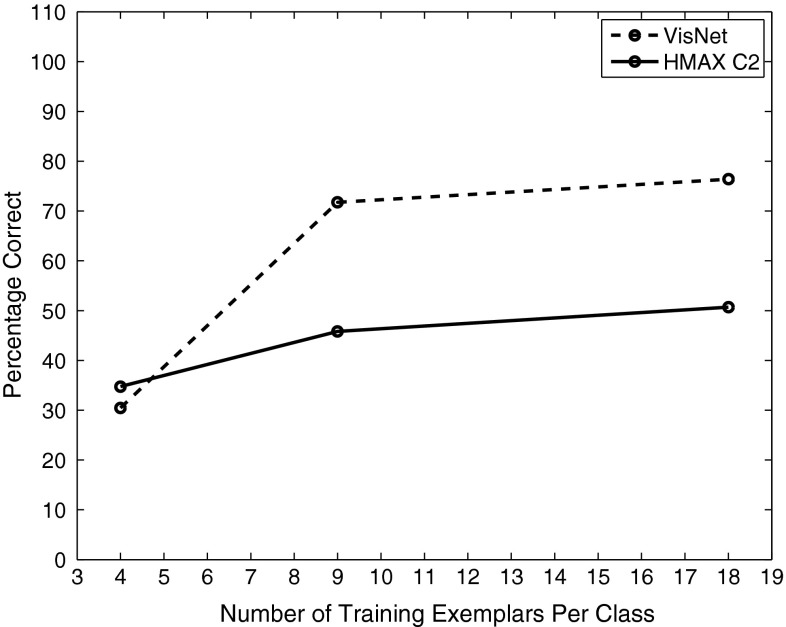
Performance of VisNet and HMAX C2 units measured by the percentage of images classified correctly on the classification task with 8 objects using the Amsterdam Library of Images dataset and measurement of performance using a pattern association network with one output neuron for each class. The training set consisted of 4 views of each object spaced $90^{\circ }$ apart; or 9 views spaced $40^{\circ }$ apart; or 18 views spaced $20^{\circ }$ apart. The test set of images was in all cases a cross-validation set of 18 views of each object spaced $20^{\circ }$ apart and offset by $10^{\circ }$ from the training set with 18 views and not including any training view. The 10 best cells from each class were used to measure the performance. Chance performance was 12.5 % correct

**Fig. 8:**
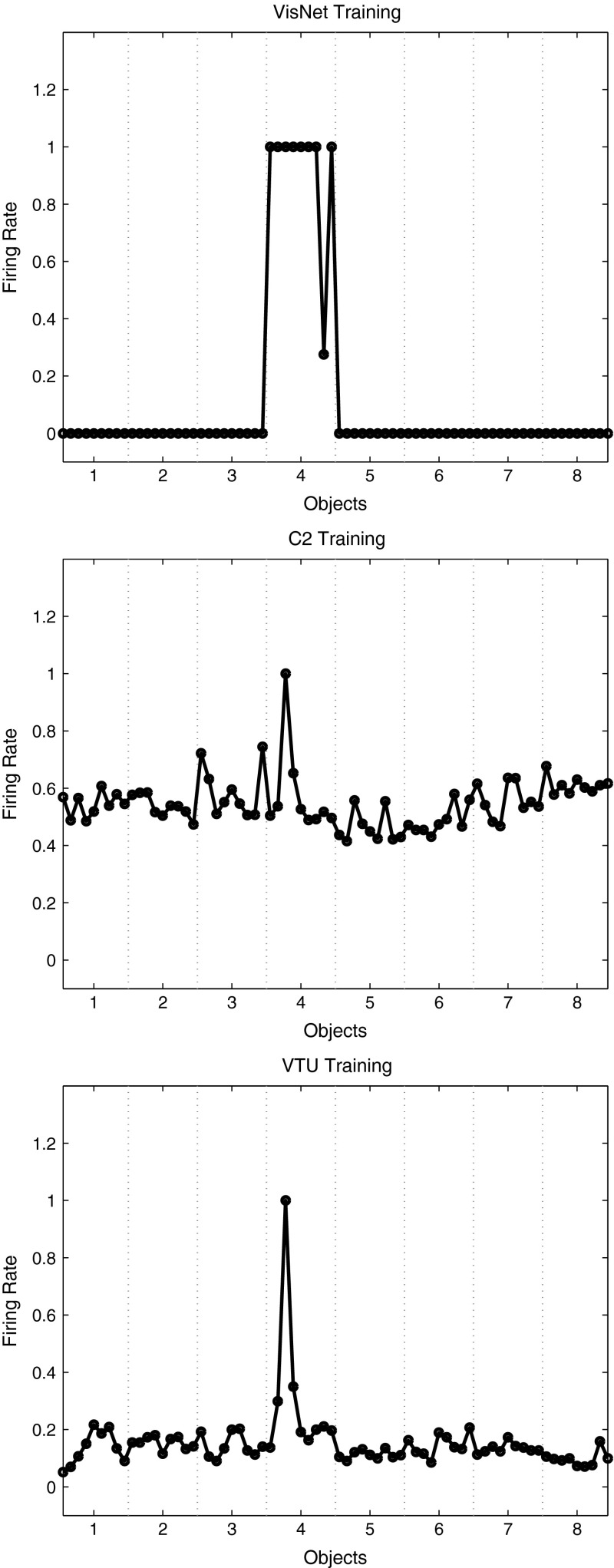
*Top* firing rate of one output layer neuron of VisNet, when trained on 8 objects from the Amsterdam Library of Images, with 9 views of each object spaced $40^{\circ }$ apart. The firing rates on the training set are shown. The neuron responded to all 9 views of object 4 (a light bulb) and to no views of any other object. The neuron illustrated was chosen to have the highest single cell stimulus-specific information about object 4 that could be decoded from the responses of the neurons to all 72 exemplars shown, as well as a high firing rate to object 4. *Middle* firing rate of one C2 unit of HMAX when trained on the same set of images. The unit illustrated was that the highest mean firing rate across views to object 4 relative to the firing rates across all stimuli and views. *Bottom* firing rate of one view-tuned unit (VTU) of HMAX when trained on the same set of images. The unit illustrated was that the highest firing rate to one view of object 4

Figure 7 shows (measuring performance with a pattern associator trained on the 10 best cells for each of the 8 classes) that VisNet performed moderately well as soon as there were even a few training images, with the coding of its outputs thus shown to be suitable for learning by a pattern association network. In a statistical control, we found that an untrained VisNet performed at 18 % correct when measured with the pattern association network compared with the 73 % correct after training with 9 exemplars that is shown in Fig. 7. HMAX performed less well than VisNet. There was some information in the output of the HMAX C2 neurons, for if a powerful linear support vector machine (SVM) was used across all output layer neurons, the performance in particular for HMAX improved, with 78 % correct for 4 training views and 93 % correct for 9 training views and 92 % correct for 18 training views (which in this case was also achieved by VisNet).

What VisNet can do here is to learn view-invariant representations using its trace learning rule to build feature analysers that reflect the similarity across at least adjacent views of the training set. Very interestingly, with 9 training images, the view spacing of the training images was $40^{\circ }$, and the test images in the cross-validation design were the intermediate views, $20^{\circ }$ away from the nearest trained view. This is promising, for it shows that enormous numbers of training images with many different closely spaced views are not necessary for VisNet. Even 9 training views spaced $40^{\circ }$ apart produced reasonable training.

We next compared the outputs produced by VisNet and HMAX, in order to assess their biological plausibility. Figure 8 Upper shows the firing rate of one output layer neuron of VisNet, when trained on 8 objects from the Amsterdam Library of Images, with 9 exemplars of each object with views spaced $40^{\circ }$ apart (set 2 described above). The firing rates on the training set are shown. The neuron responded to all 9 views of object 4 (a light bulb) and to no views of any other object. The neuron illustrated was chosen to have the highest single cell stimulus-specific information about object 4 that could be decoded from the responses of a neuron to the 9 exemplars of object 4 (as well as a high firing rate). That information was 3 bits. The mean stimulus-specific single cell information across the 5 most informative cells for each class was 2.2 bits. Figure 8 Middle shows the firing rate of one C2 unit of HMAX when trained on the same set of images. The unit illustrated was that with the highest mean firing rate across views to object 4 relative to the firing rates across all stimuli and views. The neuron responded mainly to one of the 9 views of object 4, with a small response to 2 nearby views. The neuron provided little information about object 4, even though it was the most selective unit for object 4. Indeed, the single cell stimulus-specific information for this C2 unit was 0.68 bits. The mean stimulus-specific single cell information across the 5 most informative C2 units for each class was 0.28 bits. Figure 8 Bottom shows the firing rate of one VTU of HMAX when trained on the same set of images. The unit illustrated was that with the highest firing rate to one view of object 4. Small responses can also be seen to view 2 of object 4 and to view 9 of object 4, but apart from this, most views of object 4 were not discriminated from the other objects. The single cell stimulus-specific information for this VTU was 0.28 bits. The mean stimulus-specific single cell information across the 5 most informative VTUs for each class was 0.67 bits.

The stimulus-specific single unit information measures show that the neurons of VisNet have much information in their firing rates about which object has been shown, whereas there is much less information in the firing rates of HMAX C2 units or view-tuned units. The firing rates for different views of an object are highly correlated for VisNet, but not for HMAX. This is further illustrated in Fig. 10, which shows the similarity between the outputs of the networks between the 9 different views of 8 objects produced by VisNet (top), HMAX C2 (middle), and HMAX VTUs (bottom) for the Amsterdam Library of Images test. Each panel shows a similarity matrix (based on the cosine of the angle between the vectors of firing rates produced by each object) between the 8 stimuli for all output neurons of each network. The maximum similarity is 1, and the minimal similarity is 0. The results are from the simulations with 9 views of each object spaced $40^{\circ }$ apart during training, with the testing results illustrated for the 9 intermediate views $20^{\circ }$ from the nearest trained view. For VisNet (top), it is shown that the correlations measured across the firing rates of all output neurons are very similar for all views of each object (apart from 2 views of object 1) and that the correlations with all views of every other object are close to 0.0. For HMAX C2 units, the situation is very different, with the outputs to all views of all objects being rather highly correlated, with a minimum correlation of 0.975. In addition, the similarity of the outputs produced by the different views of any given object is little more than the similarity with the views of other objects. This helps to emphasize the point that the firing within HMAX does not reflect well even a view of one object as being very different from the views of another object, let alone that different views of the same object produce similar outputs. This emphasizes that for HMAX to produce measurably reasonable performance, most of the classification needs to be performed by a powerful classifier connected to the outputs of HMAX, not by HMAX itself. The HMAX VTU firing (bottom) was more sparse ($\sigma$ was 1.0), but again the similarities between objects are frequently as great as the similarities within objects.

Figure 2 of the Supplementary Material shows that similar results were obtained for the HMAX implementation by Serre et al. (2007a).

**Fig. 9:**
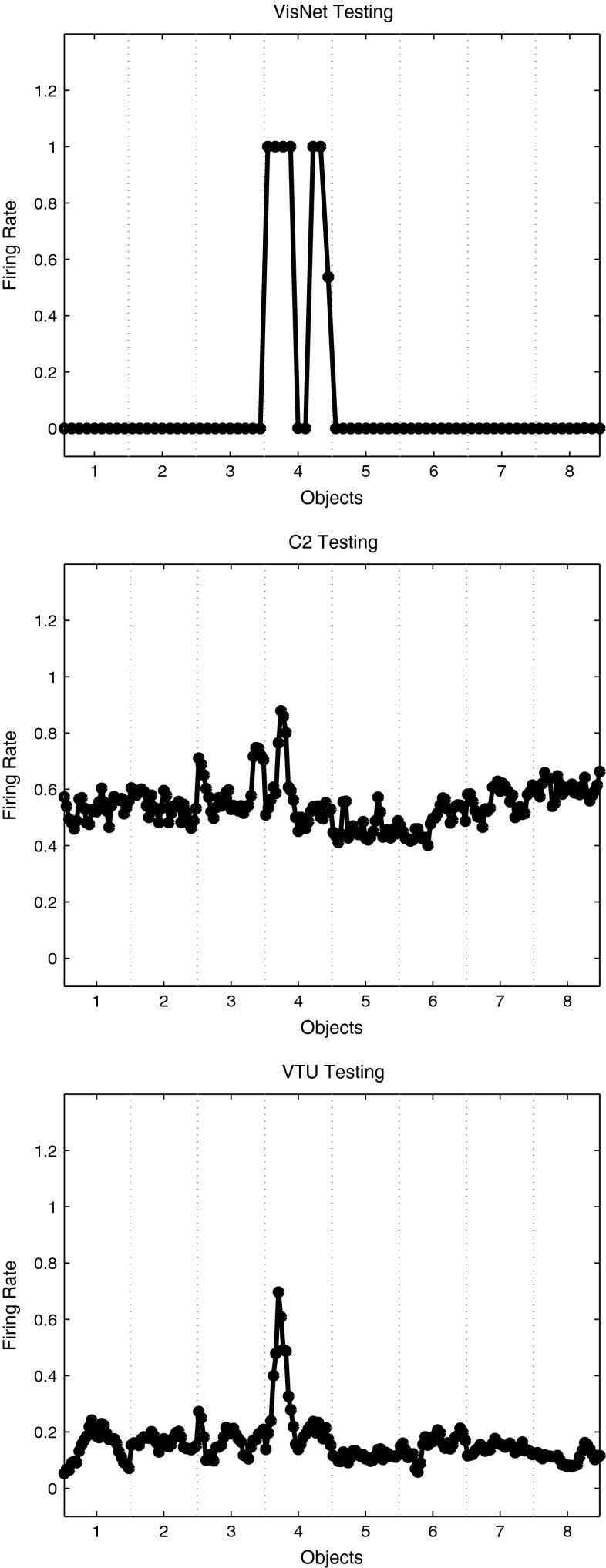
*Top* firing rate during cross-validation testing of one output layer neuron of VisNet, when trained on 8 objects from the Amsterdam Library of Images, with 9 exemplars of each object with views spaced $40^{\circ }$ apart. The firing rates on the cross-validation testing set are shown. The neuron was selected to respond to all views of object 4 of the training set, and as shown responded to 7 views of object 4 in the test set each of which was $20^{\circ }$ from the nearest training view and to no views of any other object. *Middle* firing rate of one C2 unit of HMAX when tested on the same set of images. The neuron illustrated was that the highest mean firing rate across training views to object 4 relative to the firing rates across all stimuli and views. The test images were $20^{\circ }$ away from the test images. *Bottom* firing rate of one view-tuned unit (VTU) of HMAX when tested on the same set of images. The neuron illustrated was that the highest firing rate to one view of object 4 during training. It can be seen that the neuron responded with a rate of 0.8 to the two training images (1 and 9) of object 4 that were $20^{\circ }$ away from the image for which the VTU had been selected

Experiment 2 thus shows that with the ALOI training set, VisNet can form separate neuronal representations that respond to all exemplars of each of 8 objects seen in different view transforms and that single cells can provide perfect information from their firing rates to any exemplar about which object is being presented. The code can be read in a biologically plausible way with a pattern association network, which achieved 77 % correct on the cross-validation set. Moreover, with training views spaced $40^{\circ }$ apart, VisNet performs moderately well (72 % correct) on the intermediate views ($20^{\circ }$ away from the nearest training view) (Fig. 9 Top). In contrast, C2 output units of HMAX discriminate poorly between the object classes (Fig. 9 Middle), view-tuned units of HMAX respond only to test views that are $20^{\circ }$ away from the training view, and the performance of HMAX tested with a pattern associator is correspondingly poor.

**Fig. 10:**
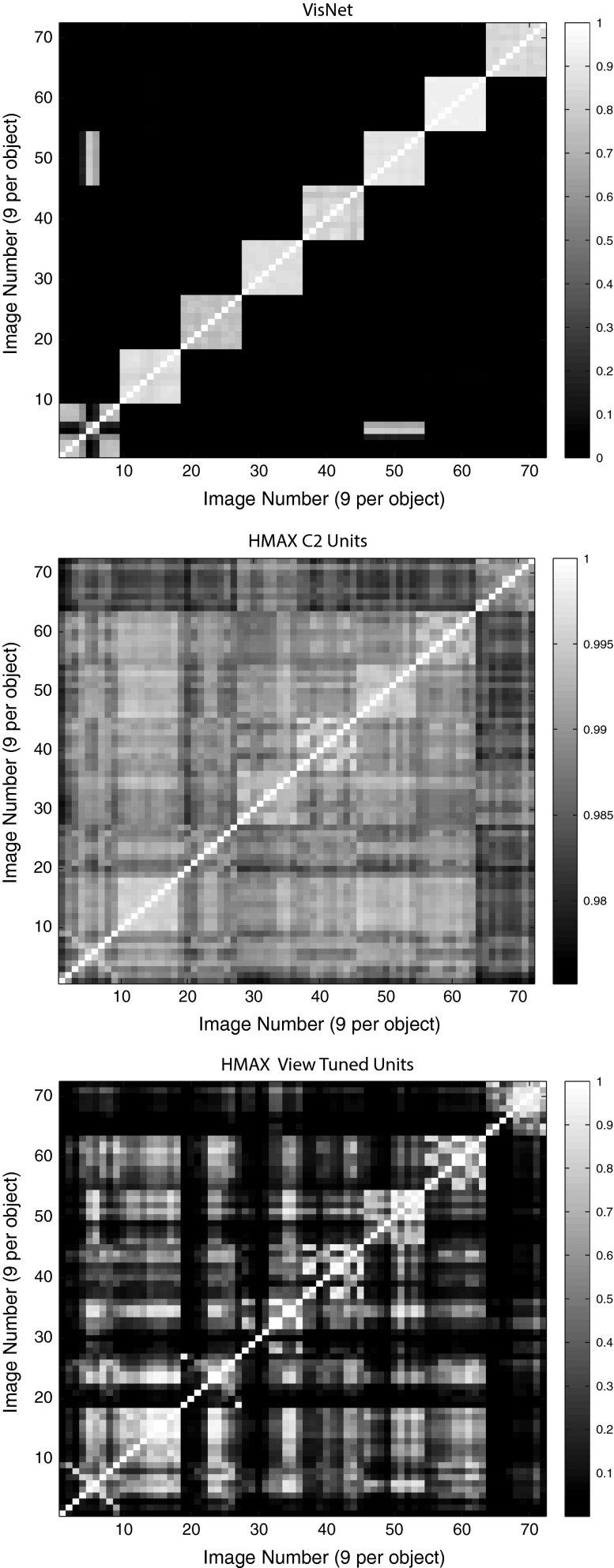
Similarity between the outputs of the networks between the 9 different views of 8 objects produced by VisNet (*top*), HMAX C2 (*middle*), and HMAX VTUs (*bottom*) for the Amsterdam Library of Images test. Each *panel* shows a similarity matrix (based on the cosine of the angle between the vectors of firing rates produced by each object) between the 8 stimuli for all output neurons of each type. The maximum similarity is 1, and the minimal similarity is 0

### Effects of rearranging the parts of an object: Experiment 3

Rearranging parts of an object can disrupt identification of the object, while leaving low-level features still present. Some face-selective neurons in the inferior temporal visual cortex do not respond to a face if its parts (e.g. eyes, nose, and mouth) are rearranged, showing that these neurons encode the whole object and do not respond just to the features or parts (Rolls et al. 1994; Perrett et al. 1982). Moreover, these neurons encode identity in that they respond differently to different faces (Baylis et al. 1985; Rolls et al. 1997a, b; Rolls and Treves 2011). We note that some other neurons in the inferior temporal visual cortex respond to parts of faces such as eyes or mouth (Perrett et al. 1982; Issa and DiCarlo 2012), consistent with the hypothesis that the inferior temporal visual cortex builds configuration-specific whole face or object representations from their parts, helped by feature combination neurons learned at earlier stages of the ventral visual system hierarchy (Rolls 1992, 2008, 2012a, 2016) (Fig. 1).

**Fig. 11:**
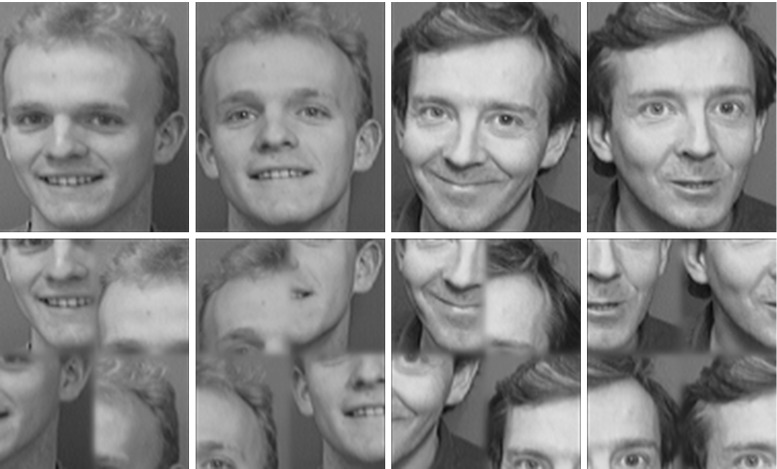
Examples of images used in the scrambled faces experiment. *Top* two of the 8 faces in 2 of the 5 views of each. *Bottom* examples of the scrambled versions of the faces

To investigate whether neurons in the output layers of VisNet and HMAX can encode the identity of whole objects and faces (as distinct from their parts, low-level features, etc.), we tested VisNet and HMAX with normal faces and with faces with their parts scrambled. We used 8 faces from the ORL database of faces (http://www.cl.cam.ac.uk/research/dtg/attarchive/facedatabase.html) each with 5 exemplars of different views, as illustrated in Fig. 11. The scrambling was performed by taking quarters of each face and making 5 random permutations of the positions of each quarter. The procedure was to train on the set of unscrambled faces and then to test how the neurons that responded best to each face then responded when the scrambled versions of the faces were shown, using randomly scrambled versions of the same eight faces each with the same set of 5 view exemplars.

**Fig. 12:**
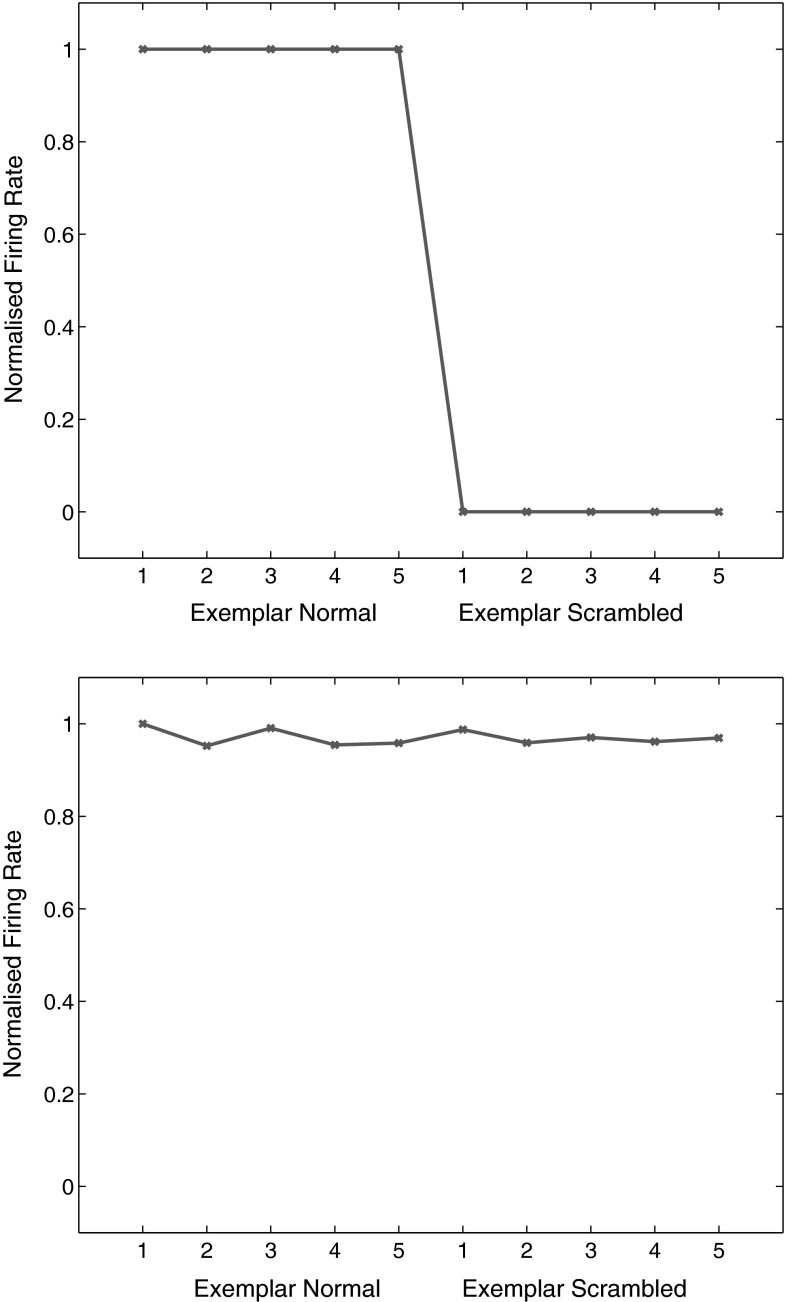
*Top* effect of scrambling on the responses of a neuron in VisNet. This VisNet layer 4 neuron responded to one of the faces after training and to none of the other 7 faces. The neuron responded to all the different view exemplars 1–5 of the unscrambled face (exemplar normal). When the same neuron was then tested with the randomly scrambled versions of the same face stimuli (exemplar scrambled), the firing rate was zero. *Bottom* effect of scrambling on the responses of a neuron in HMAX. This view-tuned neuron of HMAX was chosen to be as discriminating between the 8 face identities as possible. The neuron responded to all the different view exemplars 1–5 of the unscrambled face. When the same neuron was then tested with the randomly scrambled versions of the same face stimuli, the neuron responded with similarly high rates to the scrambled stimuli

VisNet was trained for 20 epochs and performed 100 % correct on the training set. When tested with the scrambled faces, performance was at chance, 12.5 % correct, with 0.0 bits of multiple cell information using the 5 best cells for each class. An example of a VisNet layer 4 neuron that responded to one of the faces after training is shown in Fig. 12 top. The neuron responded to all the different view exemplars of the unscrambled face (and to no other faces in the training set). When the same neuron was then tested with the randomly scrambled versions of the same face stimuli, the firing rate was zero. In contrast, HMAX neurons did not show a reduction in their activity when tested with the same scrambled versions of the stimuli. This is illustrated in Fig. 12 bottom, in which the responses of a view-tuned neuron (selected as the neuron with most selectivity between faces, and a response to exemplar 1 of one of the non-scrambled faces) were with similarly high firing rates to the scrambled versions of the same set of exemplars. Similar results were obtained for the HMAX implementation by Serre et al. (2007a) as shown in the Supplementary Material.

This experiment provides evidence that VisNet learns shape-selective responses that do not occur when the shape information is disrupted by scrambling. In contrast, HMAX must have been performing its discrimination between the faces based not on the shape information about the face that was present in the images, but instead on some lower-level property such as texture or feature information that was still present in the scrambled images. Thus VisNet performs with scrambled images in a way analogous to that of neurons in the inferior temporal visual cortex (Rolls et al. 1994).

The present result with HMAX is a little different from that reported by Riesenhuber and Poggio (1999) where some decrease in the responses of neurons in HMAX was found after scrambling. We suggest that the difference is that in the study by Riesenhuber and Poggio (1999), the responses were not of natural objects or faces, but were simplified paper-clip types of image, in which the degree of scrambling used would (in contrast to scrambling natural objects) leave little feature or texture information that may normally have a considerable effect on the responses of neurons in HMAX.

### View-invariant object recognition: Experiment 4

Some objects look different from different views (i.e. the images are quite different from different views), yet we can recognize the object as being the same from the different views. Further, some inferior temporal visual cortex neurons respond with view-invariant object representations, in that they respond selectively to some objects or faces independently of view using a sparse distributed representation (Hasselmo et al. 1989; Booth and Rolls 1998; Logothetis et al. 1995; Rolls 2012a; Rolls and Treves 2011). An experiment was designed to compare how VisNet and HMAX operate in view-invariant object recognition, by testing both on a problem in which objects had different image properties in different views. The prediction is that VisNet will be able to form by learning neurons in its output layer that respond to all the different views of one object and to none of the different views of another object, whereas HMAX will not form neurons that encode objects, but instead will have its outputs dominated by the statistics of the individual images.

**Fig. 13:**
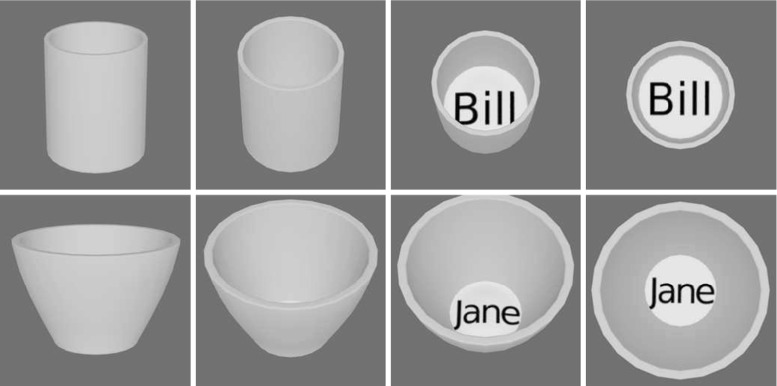
View-invariant representations of cups. The two objects, each with four views

**Fig. 14:**
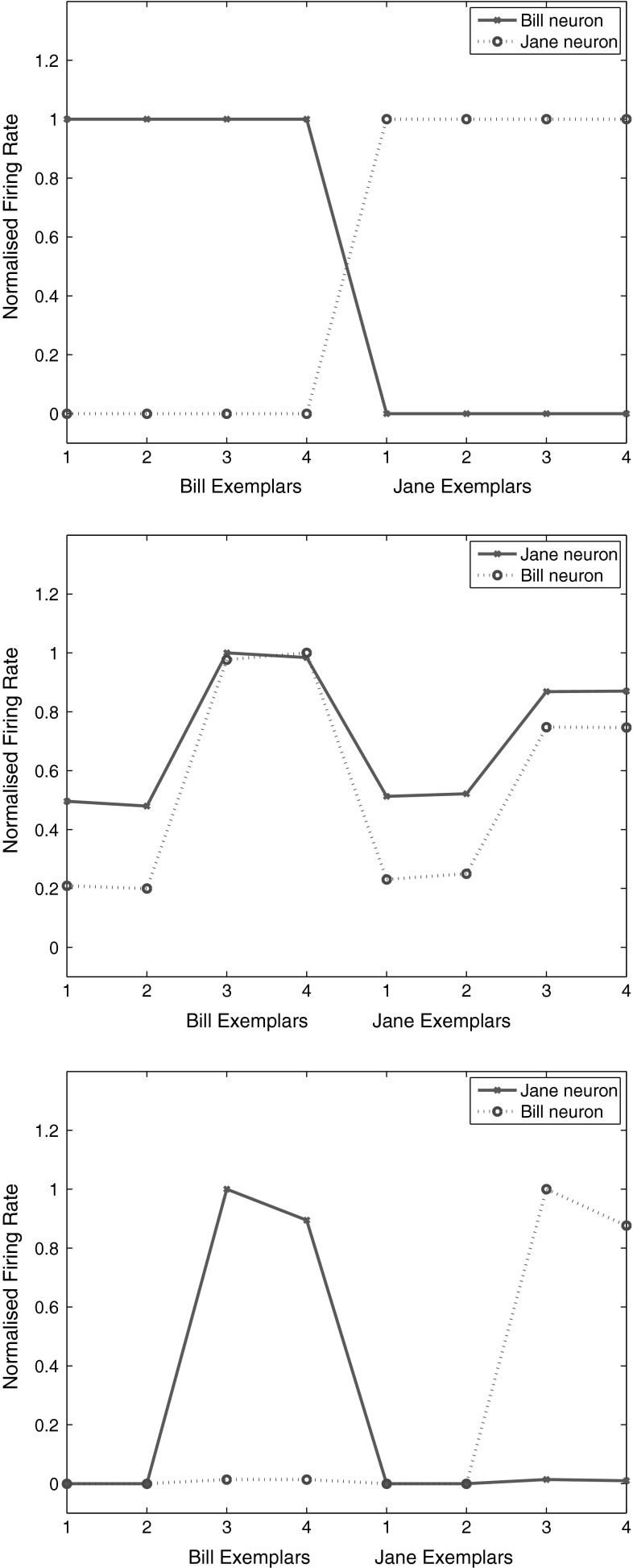
*Top* view-invariant representations of cups. Single cells in the output representation of VisNet. The two neurons illustrated responded either to all views of one cup (labelled ‘Bill’) and to no views of the other cup (labelled ‘Jane’), or vice versa. *Middle* single cells in the C2 representation of HMAX. *Bottom* single cells in the view-tuned unit output representation of HMAX

The objects used in the experiment are shown in Fig. 13. There were two objects, two cups, each with four views, constructed with Blender. VisNet was trained for 10 epochs, with all views of one object shown in random permuted sequence, then all views of the other object shown in random permuted sequence, to enable VisNet to use its temporal trace learning rule to learn about the different images that occurring together in time were likely to be different views of the same object. VisNet performed 100 % correct in this task by forming neurons in its layer 4 that responded either to all views of one cup (labelled ‘Bill’) and to no views of the other cup (labelled ‘Jane’), or vice versa, as illustrated in Fig. 14 top.

Typical most highly discriminating C2 layer neurons of HMAX are illustrated in Fig. 14 middle. The neurons did not discriminate between the objects, but instead responded more to the images of each object that contained text. This dominance by text is consistent with the fact that HMAX is up to this stage operating to a considerable extent as a set of image filters, the activity in which included much produced by the text. The performance of the C2 layer when decoded by the information analysis routines (using the 5 most object selective neurons for each class of object) was 50 % correct (where chance was 50 %), with 0.0 bits of information about which stimulus had been presented.

Typical most highly discriminating VTU (view-trained unit) layer neurons of HMAX are illustrated in Fig. 14 bottom. (Two VTUs were set up for each object during the analysis stage, one for a view of an object without text and one for a view of an object with text. $\sigma$ was set to 1.0.) The neurons did not discriminate between the objects, but instead responded much more to the images of an object that contained text. The performance of the VTU layer when decoded by the information analysis routines (using the 5 most object selective neurons for each class of object) was 50 % correct (where chance was 50 %), with 0.0 bits of information about which stimulus had been presented. A similarity matrix (based on the cosine of the angle between the vectors of firing rates produced by each stimulus) for the VTU neurons indicated that there were high correlations between the images that contained text, and high correlations between the images that did not contain text, but no correlations reflecting similar firing to all views of either object.

This experiment draws out a fundamental difference between VisNet and HMAX. The output layer neurons of VisNet can represent transform-invariant properties of objects and can form single neurons that respond to the different views of objects even when the images of the different views may be quite different, as is the case for many real-world objects when they transform in the world. Thus basing object recognition on image statistics, and categorization based on these, is insufficient for transform-invariant object recognition. VisNet can learn to respond to the different transforms of objects using the trace learning rule to capture the properties of objects as they transform in the world. In contrast, HMAX up to the C2 layer sets some of its neurons to respond to exemplars in the set of images, but has no way of knowing which exemplars may be of the same object, and no way therefore to learn about the properties of objects as they transform in the real world, showing catastrophic changes in the image as they transform (Koenderink 1990), exemplified in the example in this experiment by the new views as the objects transform from not showing to showing writing in the base of the cup. Moreover, because the C2 neurons reflect mainly the way in which all the Gabor filters respond to image exemplars, the firing of C2 neurons is typically very similar and non-sparse to different images, though if the images have very different statistics in terms of, for example, text or not, it is these properties that dominate the firing of the C2 neurons.

Similarly, the VTU neurons of HMAX are set to have synaptic strengths proportional to the firing of the C2 neurons that provide inputs to the VTUs when one view of one object is shown (Serre et al. 2007a). Because there is little invariance in the C units, many different VTUs are needed, with one for each training view or exemplar. Because the VTUs are different to each other for the different views of the same object or class, a further stage of training is then needed to classify the VTUs into object classes, and the type of learning is least squares error minimization (Serre et al. 2007a), equivalent to a delta-rule one-layer perceptron which again is not biologically plausible for neocortex (Rolls 2008). Thus HMAX does not generate invariant representations in its S–C hierarchy, and in the VTU approach uses two layers of learning after the S–C hierarchy, the second involving least squares learning, to produce classification. The representation can be more sparse than that of the C2 neurons depending on the value of $\sigma$, but nevertheless represents properties of an image and not of objects. The output of HMAX thus does not provide in general transform-invariant representations, but instead reflects statistical properties of images. Therefore, the output of HMAX must be classified by a powerful classifier such as a support vector machine, which then has to learn the whole set of outputs of the visual processing that correspond to any one object in all its transforms and views. This is biologically implausible, with pattern associators being the most typical known classifier in the cerebral cortex (Rolls 2008). In any case, because the output of C2 is so difficult to interpret by a brain-like decoder such as a pattern associator, and because VTUs by definition respond to one of perhaps many views of an object, VTUs are not generally used in more recent work with HMAX, and instead the final C layer of firing is sent directly to a support vector machine classifier (Serre et al. 2007c, a, b; Mutch and Lowe 2008).

## Discussion

### Overview of the findings on how well the properties of inferior temporal cortex neurons were met, and discussion of their significance

At the beginning of this paper, we listed some of the key properties of inferior temporal cortex (IT) neurons that need to be addressed by models of invariant visual object recognition in the ventral visual stream of the cerebral cortex. We now assess to what extent these two models account for these fundamental properties of IT neurons. This assessment is provided for these two models provided as examples and to illustrate how it may be useful for those who work with other models (e.g. Yamins et al. 2014) to assess the performance of their models against the neurophysiological data. We make these comparisons in the interest of contributing to the further development of models of how the brain solves invariant visual object recognition.

The first property is that inferior temporal visual cortex neurons show responses to objects that are typically translation, size, contrast, rotation, and in a proportion of cases view invariant, that is, they show transform invariance (Hasselmo et al. 1989; Tovee et al. 1994; Logothetis et al. 1995; Booth and Rolls 1998; Rolls 2012a). Experiment 4 with the different views of different cups shows that VisNet can solve view-invariant object recognition and that HMAX does not. VisNet solves this object recognition problem because it has a learning mechanism involving associations across time to learn that quite different views may be of the same object. HMAX has no such learning mechanism, and indeed, its performance on this task was dominated by the presence or absence of low-level image features such as whether text was visible or not. The remark might be made that HMAX is not intended to display view-invariant object recognition. But that is perhaps an important point: By having no such mechanism, HMAX does not account for a key feature of the tuning of many neurons in the inferior temporal visual cortex. In fact, VisNet uses temporo-spatial continuity to learn about all the different types of invariance and thus provides a generic approach to producing invariant representations.

The second property is that inferior temporal cortex neurons show sparse distributed representations, in which individual neurons have high firing rates to a few stimuli (e.g. objects or faces) and lower firing rates to more stimuli, in which much information can be read from the responses of a single neuron from its firing rates using, for example, dot product decoding (because the neuronal responses are high to relatively few stimuli), and in which neurons encode independent information about a set of stimuli, as least up to tens of neurons (Rolls and Tovee 1995; Rolls et al. 1997a, b; Abbott et al. 1996; Baddeley et al. 1997; Rolls 2008, 2012a; Rolls and Treves 2011). Experiment 2 shows that VisNet produces single neurons with sparse representations, in which a single neuron can respond to many exemplars of one object and to no exemplars of many other objects (Figs. 8, 9). (Although these representations are relatively binary, with the most selective neurons for an object having high firing rates to only one object and low firing rates to all other objects, other neurons that are less selective have more graded firing rate representations, and the representations can be made more graded by reducing the value of the sigmoid activation function parameter $\beta$ specified in Eq. (5)) In contrast HMAX produces neurons in its final C layer that have highly distributed representations, with even the most selective single neurons having high firing rates to almost all the stimuli (see Figs. 8, 9) for the C2 top layer neurons with the Mutch and Lowe (2008) implementation of HMAX, and Figs. 1 and 2 of the Supplementary Material for the top layer C3 neurons of the Serre et al. (2007a) version of HMAX. Consistent with this, the information could not be read from these final C layers of HMAX by a biologically plausible pattern association network using dot product decoding, but required a much more powerful support vector machine or linear least squares regressor equivalent to a delta-rule perceptron to classify these outputs of HMAX. If view-tuned units were used to read the outputs of HMAX, then these units did have a more sparse representation, but again had some responses to all the exemplars of all objects as shown in the same Figures, and as noted for the first property, did not have view-invariant representations and so again required powerful decoding to read the VTUs to classify an image as a particular object. That is, most of the work for the classification was done by the external system reading the activity of the output neurons, rather than being present in the firing of the HMAX neurons themselves. Similar conclusions about the representations produced by HMAX follow from Experiment 1 with the CalTech stimuli, though as noted below under property 4, the use of such datasets and classification into a class of object such as animal versus non-animal does not capture the fundamental property 4 of encoding information about individual faces or objects, as contrasted with classes.

A third property is that inferior temporal cortex neurons often respond to objects and not to low-level features, in that many respond to whole objects, but not to the parts presented individually, nor to the parts presented with a scrambled configuration (Perrett et al. 1982; Rolls et al. 1994). Experiment 3 showed that rearranging the parts of an object (‘scrambling’) led to no responses from VisNet neurons that responded to the whole object, showing that it implemented whole object recognition, rather than just having responses to features. This follows up with images of objects what was shown previously for VisNet with more abstract stimuli, combinations of up to four lines in different spatial arrangements to specify different shapes (Elliffe et al. 2002). In contrast, HMAX final layer neurons responded also to the scrambled images, providing an indication that HMAX does not implement shape representations of whole objects in which the parts are in the correct spatial arrangement, but instead allows features to pass from its Gabor filters to the output on the basis of which a powerful classifier might be able to specify because of the types of low-level features what class of object may be present. This may be satisfactory for low-level feature identification that might then be used to classify objects into classes using e.g. a SVM, but is hardly a basis for shape recognition, which is a key property of IT neurons. VisNet solves the shape problem by implementing a feature hierarchy in which combinations of features in the correct spatial relationship become learned at every stage of the processing, resulting in shape not low-level feature recognition (Rolls 1992, 2008, 2012a; Elliffe et al. 2002).

A fourth property is that inferior temporal cortex neurons convey information about the individual object or face, not just about a class such as face versus non-face, or animal versus non-animal (Rolls and Tovee 1995; Rolls et al. 1997a, b; Abbott et al. 1996; Baddeley et al. 1997; Rolls 2008, 2011, 2012a; Rolls and Treves 2011). This key property is essential for recognizing a particular person or object and is frequently not addressed in models of invariant object recognition, which still focus on classification into, e.g. animal versus non-animal, or classes such as hats and bears from databases such as the CalTech (Serre et al. 2007a, b; Mutch and Lowe 2008; Serre et al. 2007c; Yamins et al. 2014). It is clear that VisNet has this key property of representing individual objects, faces, etc., as is illustrated in Experiments 2, 3, and 4, and previously (Rolls and Milward 2000; Stringer and Rolls 2000, 2002; Rolls and Webb 2014; Webb and Rolls 2014; Stringer et al. 2006; Perry et al. 2006, 2010; Rolls 2012a). VisNet achieves this by virtue of its competitive learning, in combination with its trace learning rule to learn that different images are of the same object. It is unfortunate that we know little about this from previous publications with HMAX, but the results shown in Experiment 4 provide evidence that HMAX may categorize together images with similar low-level feature properties (such as the presence of text), and not perform shape recognition relevant to the identification of an individual in which the spatial arrangements of the parts are important, as shown in Experiment 3.

A fifth property is that the learning mechanism involved in invariant object recognition needs to be biologically plausible and that is likely to include a local synaptic learning rule (Rolls 2008). This is implemented in VisNet, in that the information present to alter the strength of the synapse is present in the firing of the presynaptic and postsynaptic neuron, as is shown in Eq. (1). We note that lateral propagation of weights, as used in the neocognitron (Fukushima 1980), HMAX (Riesenhuber and Poggio 1999; Mutch and Lowe 2008; Serre et al. 2007a), and convolution nets (LeCun et al. 2010), is not biologically plausible (Rolls 2008).

### Training method

One difference highlighted by these investigations is that VisNet is normally trained on images generated by objects as they transform in the world, so that view, translation, size, rotation, etc., invariant representations of objects can be learned by the network. In contrast, HMAX is typically trained with large databases of pictures of different exemplars of, for example, hats and beer mugs as in the CalTech databases, which do not provide the basis for invariant representations of objects to be learned, but are aimed at object classification. However, it is shown in Experiment 1 that VisNet can perform this object classification in a way that is comparable to HMAX. In Experiment 1, it is also shown that the activity of the output of the last layer of HMAX C neurons is very non-sparse, provides very little information in the single-neuron responses about the object class, cannot be read by biologically plausible decoding such as might be performed in the brain by a pattern association network, and requires a support vector machine (or view-tuned neurons followed by least squares learning) to learn the classification. In contrast, because of the invariance learning in VisNet, the single neurons in the sparse representation at the output provide information about which class of object was shown, and the population can be read by a biologically plausible pattern association network. VisNet thus provides a representation similar to that of neurons in the inferior temporal visual cortex (Rolls and Treves 2011; Rolls 2012a), and HMAX does not produce representations that are like those found in the inferior temporal visual cortex.

In Experiment 2, it is shown that VisNet performs well with sets of images (from the ALOI set) that provide exemplars that allow view-invariant representations to be formed. HMAX performs poorly at this type of task when assessed with biologically plausible measures, in that the C2 neurons discriminate poorly between the classes, and in that the VTU neurons generalize only to adjacent views, as there is no mechanism in HMAX to enable it to learn that quite different images may be different views of the same object. HMAX thus has to rely on powerful pattern classification mechanisms such as a support vector machine to make sense of its output representation. The difference of the output representations is also marked. Single neurons in VisNet provide considerable stimulus-specific information about which object was shown (e.g. 3 bits depending on the set size, with the maximum information being $\log _2 S$ where *S* is the number of objects in the set), in a way that is similar to that of neurons in the inferior temporal visual cortex (Tovee et al. 1993; Rolls et al. 1997b). In contrast, individual neurons in the HMAX C2 layer are poorly tuned to the stimuli and contain little stimulus-specific information about views let alone about objects. The same point applies to many other computer-based object recognition systems, including deep convolutional neural networks, namely that they have no way of learning transform invariances from systematically transformed sets of training exemplars of objects.

### Representations of the spatial configurations of the parts

In Experiment 3, it is shown that VisNet neurons do not respond to scrambled images of faces, providing evidence that they respond to the shape information about faces, and objects, and not to low-level features or parts. In contrast, HMAX neurons responded with similarly high rates to both the unscrambled and scrambled faces, indicating that low-level features including texture may be very relevant to the performance and classification produced by HMAX.

### Object representations invariant with respect to catastrophic view transformations

In Experiment 4, it is shown that VisNet can learn to recognize objects even when the view provided by the object changes catastrophically as it transforms, whereas HMAX has no learning mechanism in its S–C hierarchy that provides for this type of view-invariant learning.

Thus the approach taken by VisNet provides a model of ventral visual stream processing that produces neurons at its output layer that are very similar in their invariant representations to those found in the inferior temporal visual and that can be read by pattern association networks in brain regions such as the orbitofrontal cortex and amygdala. In contrast, the approach taken in HMAX does not lead to neurons in the output C layer that provide view-invariant representations of objects, are very non-sparse and unlike those found in visual cortical areas, and needs the major part of any object classification required to be performed by an artificial neural network such as a support vector machine. These investigations of different approaches to how the ventral visual stream can produce firing like that of neurons in the inferior temporal visual cortex that can be easily read by biologically plausible networks such as pattern associators have implications for future research and provide interesting contrasts of approaches used in biologically plausible object recognition networks with transform-invariant representations of objects and artificial neural networks required to perform pattern classification. Our main aim here of comparing these two networks is that the comparison helps highlight what a biologically plausible model of the ventral visual system in invariant visual object recognition needs to account for.

### How VisNet solves the computational problems of view-invariant representations

We provide now an account of how VisNet is able to solve the type of invariant object recognition problem described here when an image is presented to it, with more detailed accounts available elsewhere (Rolls 2008, 2012a; Wallis and Rolls 1997). VisNet is a 4-layer network with feedforward convergence from stage to stage that enables the small receptive fields present in its V1-like Gabor filter inputs of approximately $1^{\circ }$ to increase in size so that by the fourth layer a single neuron can potentially receive input from all parts of the input space (Fig. 1). The feedforward connections between layers are trained by competitive learning, which is an unsupervised form of learning (Rolls 2008), that allows neurons to learn to respond to feature combinations. As one proceeds up though the hierarchy, the feature combinations become combinations of feature combinations (see Rolls 2008 Fig. 4.20 and Elliffe et al. 2002). Local lateral inhibition within each layer allows each local area within a layer to respond to and learn whatever is present in that local region independently of how much information and contrast there may be in other parts of a layer, and this, together with the non-linear activation function of the neurons, enables a sparse distributed representation to be produced. In the sparse distributed representation, a small proportion of neurons is active at a high rate for the input being presented, and most of the neurons are close to their spontaneous rate, and this makes the neurons of VisNet (Rolls 2008, 2012a) very similar to those recorded in the visual system (Rolls and Treves 2011; Rolls 2008; Tovee et al. 1993; Rolls et al. 1997a, b; Abbott et al. 1996). A key property of VisNet is the way that it learns whatever can be learned at every stage of the network that is invariant as an image transforms in the natural world, using the temporal trace learning rule. This learning rule enables the firing from the preceding few items to be maintained, and given the temporal statistics of visual inputs, these inputs are likely to be from the same object. (Typically primates including humans look at one object for a short period during which it may transform by translation, size, isomorphic rotation, and/or view, and all these types of transform can therefore be learned by VisNet.) Effectively, VisNet uses as a teacher the temporal and spatial continuity of objects as they transform in the world to learn invariant representations. (An interesting example is that representations of individual people or objects invariant with respect to pose (e.g. standing, sitting, walking) can be learned by VisNet, or representations of pose invariant with respect to the individual person or object can be learned by VisNet depending on the order in which the identical images are presented during training (Webb and Rolls 2014).) Indeed, we developed these hypotheses (Rolls 1992, 1995, 2012a; Wallis et al. 1993) into a model of the ventral visual system that can account for translation, size, view, lighting, and rotation invariance (Wallis and Rolls 1997; Rolls and Milward 2000; Stringer and Rolls 2000, 2002, 2008; Rolls and Stringer 2001, 2006, 2007; Elliffe et al. 2002; Stringer et al. 2006, 2007; Perry et al. 2006, 2010; Rolls 2008, 2012a). Consistent with the hypothesis, we have demonstrated these types of invariance (and spatial frequency invariance) in the responses of neurons in the macaque inferior temporal visual cortex (Rolls and Baylis 1986; Rolls et al. 1985, 1987, 2003; Hasselmo et al. 1989; Tovee et al. 1994; Booth and Rolls 1998). Moreover, we have tested the hypothesis by placing small 3D objects in the macaque’s home environment and showing that in the absence of any specific rewards being delivered, this type of visual experience in which objects can be seen from different views as they transform continuously in time to reveal different views leads to single neurons in the inferior temporal visual cortex that respond to individual objects from any one of the several different views, demonstrating the development of view-invariance learning (Booth and Rolls 1998). (In control experiments, view-invariant representations were not found for objects that had not been viewed in this way.) The learning shown by neurons in the inferior temporal visual cortex can take just a small number of trials (Rolls et al. 1989). The finding that temporal contiguity in the absence of reward is sufficient to lead to view-invariant object representations in the inferior temporal visual cortex has been confirmed (Li and DiCarlo 2008, 2010, 2012). The importance of temporal continuity in learning invariant representations has also been demonstrated in human psychophysics experiments (Perry et al. 2006; Wallis 2013). Some other simulation models are also adopting the use of temporal continuity as a guiding principle for developing invariant representations by learning (Wiskott and Sejnowski 2002; Wiskott 2003; Franzius et al. 2007; Einhauser et al. 2005; Wyss et al. 2006) (see review by Rolls 2012a), and the temporal trace learning principle has also been applied recently (Isik et al. 2012) to HMAX (Riesenhuber and Poggio 2000; Serre et al. 2007b) and to V1 (Lies et al. 2014).

VisNet is also well adapted to deal with real-world object recognition. If different backgrounds are present during testing, this does not disrupt the identification of particular objects previously trained, because the different backgrounds are not associated with the object to be recognized. This process is helped by the fact that the responses of inferior temporal cortex neurons shrink from approximately 78$^{\circ }$ in diameter in a scene with one object on a blank background, to approximately 22$^{\circ }$ in a complex natural scene (Rolls et al. 2003). This greatly facilitates processing in the ventral visual cortical stream object recognition system, for it means that it is much more likely that there is only one object or a few objects to be dealt with at the fovea that need to be recognized (Rolls et al. 2003; Rolls and Deco 2006). The mechanism for the shrinking of the receptive fields of inferior temporal cortex neurons in complex natural scenes is probably lateral inhibition from nearby visual features and objects, which effectively leave a neuron sensitive to only the peak of the receptive field, which typically includes the fovea because of its greater cortical magnification factor for inferior temporal cortex neurons (Trappenberg et al. 2002). Moreover, for similar reasons, VisNet can learn to recognize individual objects if they presented simultaneously with other objects chosen randomly (Stringer and Rolls 2008; Stringer et al. 2007).

### Approach taken by HMAX

We now compare this VisNet approach to invariant object recognition to the approach of HMAX, another approach that seeks to be biologically plausible (Riesenhuber and Poggio 2000; Serre et al. 2007c, a, b; Mutch and Lowe 2008), which is a hierarchical feedforward network with alternating simple cell-like (S) and complex cell-like (C) layers inspired by the architecture of the primary visual cortex, V1. The simple cell-like layers respond to a similarity function of the firing rates of the input neuron to the synaptic weights of the receiving neuron (used as an alternative to the more usual dot product) and the complex cells to the maximum input that they receive from a particular class of simple cell in the preceding layer. The classes of simple cell are set to respond maximally to a random patch of a training image (by presenting the image, and setting the synaptic weights of the S cells to be the firing rates of the cells from it receives), and are propagated laterally, that is, there are exact copies throughout a layer, which is of course a non-local operation and not biologically plausible. The hierarchy receives inputs from Gabor-like filters (which is like VisNet). The result of this in HMAX is that in the hierarchy, there is no learning of invariant representations of objects and that the output firing in the final C layer (for example the second C layer in a four-layer S1–C1–S2–C2 hierarchy) is high for almost all neurons to most stimuli, with almost no invariance represented in the output layer of the hierarchy, in that two different views of the same object may be as different as a view of another object, measured using the responses of a single neuron or of all the neurons. The neurons in the output C layer are thus quite unlike those in VisNet or in the inferior temporal cortex, where there is a sparse distributed representation, and where single cells convey much information in their firing rates, and populations of single cells convey much information that can be decoded by biologically plausible dot product decoding (Rolls and Treves 2011; Rolls 2008; Tovee et al. 1993; Rolls et al. 1997a, b; Abbott et al. 1996) such as might be performed by a pattern association network in the areas that receive from the inferior temporal visual cortex, such as the orbitofrontal cortex and amygdala (Rolls 2008, 2012a, 2014; Rolls and Treves 2011). HMAX therefore must resort to a powerful classification algorithm, in practice typically a support vector machine (SVM), which is not biologically plausible, to learn to classify all the outputs of the final layer that are produced by the different transforms of one object to be of the same object, and different to those of other objects. Thus HMAX does not learn invariant representations by its output layer of the S–C hierarchy, but instead uses a SVM to perform the classification that the SVM is taught. This is completely unlike the output of VisNet and of inferior temporal cortex neuron firing, which by responding very similarly in terms of firing rate to the different transforms of an object show that the invariance has been learned in the hierarchy (Rolls 2008, 2012a; Hasselmo et al. 1989; Booth and Rolls 1998).

Another way that the output of HMAX may be assessed is by the use of view-tuned units (VTUs), each of which is set to respond to one view of a class or object by setting its synaptic weights from each C unit to the value of the firing of the C unit to one view or exemplar of the object or class (Serre et al. 2007a). We note that this itself is not a biologically plausible operation, for it implies a teacher for each VTU to inform it how to respond, and then adjustment of the synaptic weights to the VTU to achieve this. Because there is little invariance in the C units, many different VTUs are needed, with one for each training view or exemplar. Because the VTUs are different to each other for the different views of the same object or class, a further stage of training is then needed to classify the VTUs into object classes, and the type of learning is least squares error minimization (Serre et al. 2007a), equivalent to a delta-rule one-layer perceptron which again is not biologically plausible for neocortex (Rolls 2008). Thus HMAX does not generate invariant representations in its S–C hierarchy, and in the VTU approach uses two layers of learning after the S–C hierarchy, the second involving least squares learning, to produce classification. This is unlike VisNet, which learns invariant representations in its hierarchy by self-organization, and produces view-invariant neurons (similar to those for faces (Hasselmo et al. 1989) and objects (Booth and Rolls 1998) in the inferior temporal visual cortex) that can be read by a biologically plausible pattern associator (Rolls 2008, 2012a). In another approach, Biederman and colleagues have shown that HMAX does not show the advantage in psychophysical performance and in the activations of area LO that is related to viewpoint invariant or nonaccidental properties (e.g. straight vs. curved), than metric properties (e.g. degree of curvature) of simple shapes.

Another difference of HMAX from VisNet is in the way that VisNet is trained, which is a fundamental aspect of the VisNet approach. HMAX has traditionally been tested with benchmarking databases such as the CalTech-101 and CalTech-256 (Griffin et al. 2007) in which sets of images from different categories are to be classified. The Caltech-256 dataset is comprised of 256 object classes made up of images that have many aspect ratios and sizes and differ quite significantly in quality (having being manually collated from web searches). The objects within the images show significant intra-class variation and have a variety of poses, illumination, scale, and occlusion as expected from natural images. A network is supposed to classify these correctly into classes such as hats and beer mugs (Rolls 2012a). The problem is that examples of each class of object transforming continuously though different positions on the retina, size, isomorphic rotation, and view are not provided to help the system learn about how a given type of object transforms in the world. The system just has to try to classify based on a set of often quite different exemplars that are not transforms of each other. Thus a system trained in this way is greatly hindered in generating transform-invariant representations by the end of the hierarchy, and such a system has to rely on a powerful classifier such as a SVM to perform a classification that is not based on transform invariance learned in the hierarchical network. In contrast, VisNet is provided during training with systematic transforms of objects of the type that would be seen as objects transform in the world and has a well-posed basis for learning invariant representations. It is important that with VisNet, the early layers may learn what types of transform can be produced in small parts of the visual field by different classes of object, so that when a new class of object is introduced, rapid learning in the last layer and generalization to untrained views can occur without the need for further training of the early layers (Stringer and Rolls 2002).

### Some other approaches to invariant visual object recognition

Some other approaches to biologically plausible invariant object recognition are being developed with hierarchies that may be allowed unsupervised learning (Pinto et al. 2009; DiCarlo et al. 2012; Yamins et al. 2014). For example, a hierarchical network has been trained with unsupervised learning and with many transforms of each object to help the system to learn invariant representations in an analogous way to that in which VisNet is trained, but the details of the network architecture are selected by finding parameter values for the specification of the network structure that produce good results on a benchmark classification task (Pinto et al. 2009). However, formally these are convolutional networks, so that the neuronal filters for one local region are replicated over the whole of visual space, which is computationally efficient but biologically implausible. Further, a general linear model is used to decode the firing in the output level of the model to assess performance, so it is not clear whether the firing rate representations of objects in the output layer of the model are very similar to that of the inferior temporal visual cortex. In contrast, with VisNet (Rolls and Milward 2000; Rolls 2012a) the information measurement procedures that we use (Rolls et al. 1997a, b) are the same as those used to measure the representation that is present in the inferior temporal visual cortex (Rolls and Treves 2011; Tovee et al. 1993; Rolls and Tovee 1995; Tovee and Rolls 1995; Abbott et al. 1996; Rolls et al. 1997a, b, 2004, 2006; Baddeley et al. 1997; Treves et al. 1999; Panzeri et al. 1999; Franco et al. 2004, 2007; Aggelopoulos et al. 2005).

### Properties of inferior temporal cortex neurons that need to be addressed by models in visual invariant object recognition

One of the important points made here is that there are a number of crucial properties of inferior temporal cortex (IT) neurons that need to be accounted for by biologically plausible models. These properties include the sparse distributed coding in which individual neurons have high firing rates to a few objects and gradually smaller responses to other stimuli. This allows much information to be read from the responses of a single neuron, or from several neurons with the information represented approximately independently for at least a limited number of neurons (Rolls and Treves 2011; Rolls 2012a; Tovee et al. 1993; Rolls and Tovee 1995; Abbott et al. 1996; Rolls et al. 1997a, b; Treves et al. 1999). This is a general property of cortical encoding and is important in the operation of associative neural networks that receive from structures such as the inferior temporal visual cortex (Rolls 2008, 2016; Rolls and Treves 2011). This is shown here to be a property of VisNet, but not of HMAX. Another property is that some IT neurons respond to parts of objects, and some only to the whole object (Perrett et al. 1982). The latter was shown here to be a property of VisNet but not HMAX. Another property is view invariance, shown by some but not all neurons in IT (Hasselmo et al. 1989; Booth and Rolls 1998), which was shown to be a property of VisNet but not HMAX. Indeed, much more transform invariance than this must be shown by a model to account for the properties of IT neurons, including translation invariance (with 70$^{\circ }$ receptive fields shrinking to approximately 15$^{\circ }$ in complex scenes), size, contrast, and spatial frequency invariance, all properties of VisNet (Rolls 2012a; Rolls and Baylis 1986; Rolls et al. 1985, 1987, 2003; Tovee et al. 1994; Logothetis et al. 1995; Booth and Rolls 1998; Trappenberg et al. 2002; Aggelopoulos and Rolls 2005). An implication is that there is very much more to testing and assessing a good model of IT performance than measuring the similarity structure of the representations of images of objects, human faces, animal faces, body parts, etc., produced by different including non-biologically plausible approaches to object recognition including deep neural networks (Khaligh-Razavi and Kriegeskorte 2014; Cadieu et al. 2014, 2013). Indeed, these measures of similarity are likely to benefit from supervised training, as has been found (Khaligh-Razavi and Kriegeskorte 2014), whereas the similarity structure of models such as VisNet that utilizes a temporal trace rule will depend on the exact similarity structure of the input across time, which needs to be taken into account in such assessments. Moreover, analysing the similarity structure of model and IT representations for classes of object does not address fundamental issues of IT encoding that IT neurons convey much information about which particular face is being shown (Rolls and Treves 2011; Rolls 2012a; Tovee et al. 1993; Rolls and Tovee 1995; Abbott et al. 1996; Rolls et al. 1997a, b; Treves et al. 1999) not just about whether it is a human or animal face or another category (Khaligh-Razavi and Kriegeskorte 2014; Cadieu et al. 2014, 2013). The present research thus emphasizes that there are a number of key properties of IT neurons that need to be taken into account in assessing how well a model accounts for the properties of IT neurons.

### Comparison with computer vision approaches to not only classification of objects but also identification of the individual

We turn next to compare the operation of VisNet, as a model of cerebral cortical mechanisms involved in view-invariant object identification, with artificial, computer vision, approaches to object identification. However, we do emphasize that our aim in the present research is to investigate how the cerebral cortex operates in vision, not how computer vision attempts to solve similar problems. Within computer vision, we note that many approaches start with using independent component analysis (ICA) (Kanan 2013), principal component analysis (PCA) (Cottrell and Hsaio 2011), sparse coding (Kanan and Cottrell 2010), and other mathematical approaches (Larochelle and Hinton 2010) to derive what may be suitable ‘feature analysers,’ which are frequently compared to the responses of V1 neurons. Computer vision approaches to object identification then may take combinations of these feature analysers and perform statistical analyses using computer-based algorithms that are not biologically plausible such as Restricted Boltzmann Machines (RBMs) on these primitives to statistically discriminate different objects (Larochelle and Hinton 2010). Such a system does not learn view-invariant object recognition, for the different views of an object may have completely different statistics of the visual primitives, yet are the different views of the same object. (Examples might include frontal and profile views of faces, which are well tolerated for individual recognition by some inferior temporal cortex neurons (Hasselmo et al. 1989); very different views of 3D object which are identified correctly as the same object by IT neurons after visual experience with the objects to allow for view-invariant learning (Booth and Rolls 1998); and many man-made tools and objects which may appear quite different in 2D image properties from different views.) Part of the difficulty of computer vision lay in attempts to parse a whole scene at one time (Marr 1982). However, the biological approach is to place the fovea on one part of a scene, perform image analysis / object identification there, and then move the eyes to fixate a different location in a scene (Rolls et al. 2003; Trappenberg et al. 2002; Rolls and Webb 2014). This is a divide-and-conquer strategy used by the real visual system, to simplify the computational problem into smaller parts performed successively, to simplify the representation of multiple objects in a scene, and to facilitate passing the coordinates of a target object for action by using the coordinates of the object being fixated (Ballard 1990; Rolls et al. 2003; Rolls and Deco 2002; Aggelopoulos and Rolls 2005; Rolls 2008, 2012a). This approach has now been adopted by some computer vision approaches (Denil et al. 2012; Kanan 2013; Kanan and Cottrell 2010). We note that non-biologically plausible approaches to object vision are important in assessing how different types of system operate with large numbers of training and test images (Khaligh-Razavi and Kriegeskorte 2014; Cadieu et al. 2014), and that there are attempts to make multilayer error correction networks more biologically plausible (O’Reilly and Munakata 2000; Balduzzi et al. 2014), but that many of these systems are far from being biological plausible. Biologically plausible systems for object recognition need to have not only the properties described here, but also mechanisms that use a local learning rule, no separate teacher for each output neuron in a supervised learning scheme, and no lateral copying of weights (Rolls 2016). Moreover, understanding how the brain operates is important not only in its own right, but also for its implications for understanding disorders of brain function (Rolls 2008, 2012b, 2016).

### Outlook: some properties of inferior temporal cortex neurons that need to be addressed by models of ventral visual stream visual invariant object recognition

The analyses described in this paper are intended to highlight some properties that models of visual object recognition in the brain in the ventral visual stream need to achieve if they are to provide an account of its functions in invariant visual object recognition, with the criteria being identified by the responses of neurons with transform-invariant representations that are found in the inferior temporal visual cortex (Rolls 2008, 2012a, 2016). First, the formation of single neurons with translation, view and rotation invariance needs to be accounted for. It is not sufficient to use a powerful decoder after the model network to achieve the required performance, instead of invariance being represented by the neurons themselves in the model of the ventral visual system. An important implication for future research is that the training set of stimuli needs to include different views of the same object and not collections of images of objects in the same class. Indeed, an important distinction is that much of what is represented in the inferior temporal visual cortex is about the invariant representation of different objects, so that individual objects or faces can be recognized from different views (Booth and Rolls 1998; Hasselmo et al. 1989), rather than just knowing that the object is a face as in a classification task. Second, the neuronal representation should be in a sparse distributed form in which much information can be read from the responses of single neurons (Rolls et al. 1997b). Third, the information should be represented approximately independently by different neurons, as least up to tens of neurons (Rolls et al. 1997a). Fourth, the neuronal representation needs to be decodable by a biologically plausible network such as a pattern association network that uses dot product decoding, which is biologically plausible for neurons (Rolls 2008; Rolls et al. 1997a; Rolls and Treves 2011). The reason why the representation is in this form in the inferior temporal visual cortex is, it is postulated, because the inferior temporal visual cortex projects directly to brain areas such as the orbitofrontal cortex and amygdala that perform pattern associations of these representations with, for example, reinforcers such as taste and touch (Rolls 2008, 2014). Fifth, the network of ventral visual stream processing needs to implement learning, for different views of an object may look very different, yet single neurons do respond to these different views (Booth and Rolls 1998; Hasselmo et al. 1989), as is required for the appropriate associations to be output by the next stages of pattern association processing (Rolls 2008, 2014). This paper has highlighted these properties. Further properties include how top-down selective attention can usefully bias the object recognition system (with a model of how this has been implemented for VisNet described previously by Deco and Rolls 2004); how cortico-cortical backprojections implement recall [with models described previously (Rolls 1989; Treves and Rolls 1994; Rolls 2008; Kesner and Rolls 2015; Rolls 2015)] [and this has implications for other possible functions that might be proposed in models of vision for backprojections (Rolls 2008, 2016)]; and how different systems scale up to deal with large numbers of objects.

### Conclusions

In conclusion, in this paper we have compared for the first time two leading approaches to object identification in the ventral visual system. We have shown how producing biologically plausible representations that are similar to those of primate inferior temporal cortex neurons is an important criterion for whether a model is successful as a model of the process. By this criterion, VisNet is biologically plausible, and HMAX is not (Experiment 1). The findings have important implications for future research, for this criterion will be important to bear in mind in developing models and theories of how the ventral visual system operates in invariant visual object recognition in future. Moreover, it is important to emphasize that neurons in the inferior temporal visual cortex provide representations suitable for the identification of individual objects, such as the face of a single individual seen from different views, and not just classification of objects such as hats, beer mugs, and umbrellas. We have also shown (Experiment 2) that there are advantages to training with training sets that provide the information for view-invariant representations of objects to be learned, rather than trying to perform classification of images as certain types of object just by seeing random exemplars of the objects in random views, which invites pattern classification based on features relevant to a class, instead of facilitating invariant representations of objects to be learned. The latter, as implemented in VisNet, provides a foundation for objects to be recognized correctly when they are shown in what can be quite different views, which is a property reflected by the responses of some neurons in the primate ventral visual pathways, in regions that include the inferior temporal visual cortex (Rolls 2008, 2012a). Another important implication is that a theory and model of the ventral visual system must be able to account for object shape recognition, not just recognition based on features or parts, as tested by scrambling the parts of objects (Experiment 3). Finally, in Experiment 4 we showed that some objects that undergo catastrophic feature changes as they transform into different views cannot be correctly categorized by systems that depend on features in an image, such as HMAX, but can be correctly identified by systems such as VisNet that can learn associations across time as objects transform naturally in time by using a synaptic learning rule with a short-term temporal trace. These findings and the conceptual points that we make have clear implications for what needs to be solved by future models of invariant visual object recognition in the ventral cortical visual stream. Moreover, the research described has clear implications for ways in which these computational problems may be solved in the ventral visual stream cortical areas.

## Electronic supplementary material

Supplementary material 1 (pdf 85 KB)
