# Supplementary material for: Invariant visual object recognition: biologically plausible approaches
Source: Biol Cybern. 2015 Sep 3;109:505–35. doi: 10.1007/s00422-015-0658-2 (PMC4572081; doi:10.1007/s00422-015-0658-2)
Supplement: Supplementary file 1 — Supplementary material 1 (pdf 85 KB) [file 422_2015_658_MOESM1_ESM.pdf]

Invariant visual object recognition: biologically plausible approaches Supplementary Material

Leigh Robinson (1) and Edmund T Rolls (1,2)
(1) University of Warwick, Department of Computer Science, Coventry, UK
and (2) Oxford Centre for Computational Neuroscience, Oxford, UK
www.oxcns.org
Edmund.Rolls@oxcns.org

1 HMAX evaluated with the version of Serre, Oliva and Poggio 2007

To check that the results described in the paper for the version of HMAX described by Mutch & Lowe (2008) are not peculiar to that version, we also performed tests of the representations provided by the version of HMAX described by Serre, Oliva & Poggio (2007), for both of which the code is available at http://cbcl.mit.edu/jmutch/cns/index.html#hmax.

The version described by Serre, Oliva & Poggio (2007) has an additional S-C layer, termed S3 and C3, in which S3 (with 10^4 units) is supposed to represent the posterior inferior temporal cortex, and C3 (with 10^3 units) the anterior inferior temporal visual cortex. The S2 and S3 units were set up in the way that they describe, by using random patches of a universal feature set (i.e. samples from the Caltech 256 image set). The C2 and C3 units responded with the max function in the way described by these authors in in the main text. We measured the performance of the C3 layer, which (just as the C2 layer of the Mutch and Lowe 2007 version) sees the whole of the input. We also measured the performance of an S4 layer which is set up to have View Tuned Units by forcing each VTU to respond to one training exemplar by setting its weights from the C3 layer to achieve this. The so-called bypass connections were not modelled, for they have inputs only to the S4 units, and the measure of performance is not markedly dependent on these bypass connections, as described by Serre et al. (2007). We used 10 S4 units per class, which corresponds to a reasonable fraction of the training exemplars, as originally implemented by Serre et al. (2007).

With this version of the architecture, we repeated Experiment 1 of the main paper, and found that the results were similar, in that neither the C3 units nor the S4 units were highly tuned to have high firing rates to even some images of one class but not the other. To illustrate this, responses of the most highly tuned C3 and S4 unit are shown in Fig. 1. For the C3 neurons, the average information from the best five single units was 0.16 bits. For the S4 VTUs, the average information from the best five single units was 0.06 bits. Nevertheless, the network we ran did perform well when tested with a powerful decoder, in that when a linear least squares classifier was implemented to decode the whole population of C3 outputs, 77% correct accuracy was confirmed, consistent with the performance levels that can be obtained with powerful decoding Serre et al. (2007).

To confirm that this result was not dependent on the choice of this Caltech 256 pair of images, we performed a further investigation in which we trained the Serre et al. (2007) version of HMAX on the animal vs non-animal categorisation task used by Serre et al. (2007). We used the same dataset (using for example 30 training exemplars of each class and 120 test exemplars of each class), and found that again neither the C3 units nor the S4 units were highly tuned to have high firing rates to even some images of one class but not the other. To illustrate this, responses of the most highly tuned C3 and S4 units are shown in Fig. 2. (We note that by selecting the units to illustrate with the highest single cell information about a class of stimulus, and the random variation between the large numbers of units from which the selection is being made, the selected units may because of random variation appear to show some evidence for classification that is not general for the population of units.) The single cell stimulus-specific information averaged across the best 5 C3 units on the testing images was very low, 0.1 bits. This further confirms the conclusions about the tuning of units in HMAX from Experiments 1 and 2, that the HMAX output units are highly distributed across an image class, and do not as single neurons discriminate well between the classes. Nevertheless, the network we ran did perform as described by Serre et al. (2007), in that when a linear least squares classifier was implemented to decode the whole population of C3 outputs, 81% correct accuracy was confirmed. These additional simulations thus support the points made in the main text.

With the version of the architecture implemented by Serre, Oliva & Poggio (2007), we also repeated Experiment 3 of the main paper with the same scrambled images, and confirmed the result that HMAX responded to the scrambled images after training on the unscrambled images, unlike shape-sensitive neurons in the inferior temporal visual cortex and unlike VisNet.

References

Geusebroek, J.-M., Burghouts, G. J. & Smeulders, A. W. M. (2005). The Amsterdam Library of Object Images, *International Journal of Computer Vision* **61**: 103–112.

Griffin, G., Holub, A. & Perona, P. (2007). The Caltech-256, *Caltech Technical Report* pp. 1–20.

Mutch, J. & Lowe, D. G. (2008). Object class recognition and localization using sparse features with limited receptive fields, *International Journal of Computer Vision* **80**: 45–57.

Serre, T., Oliva, A. & Poggio, T. (2007). A feedforward architecture accounts for rapid categorization, *Proceedings of the National Academy of Sciences* **104**: 6424–6429.

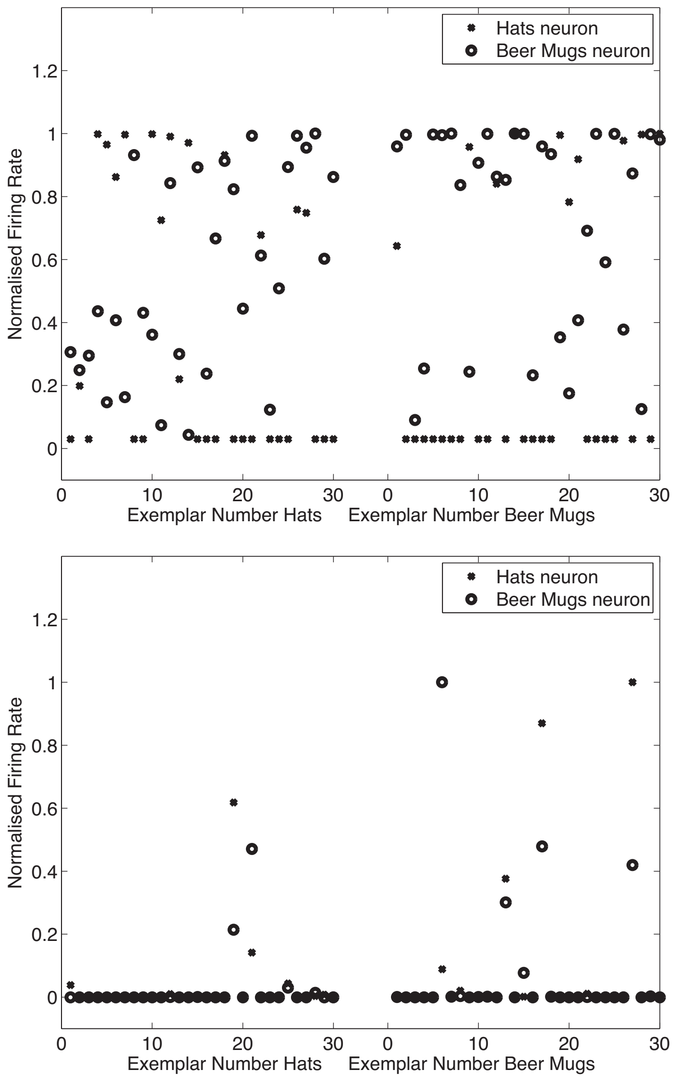

Figure 1: Top: Firing rate of two C3 units of HMAX in the Serre, Oliva and Poggio 2007 version when tested on two of the classes, beer mugs and hats, from the Caltech 256. Bottom: Firing rate of two View Tuned Unit corresponding to S4 of HMAX when tested on two of the classes, hats (solid line) and beer mugs (dashed line), from the Caltech 256. The neurons chosen were those with the highest single cell information that could be decoded from the responses of a neuron to 15 exemplars of each of the 2 objects (as well as a high firing rate) in the cross-validation design.

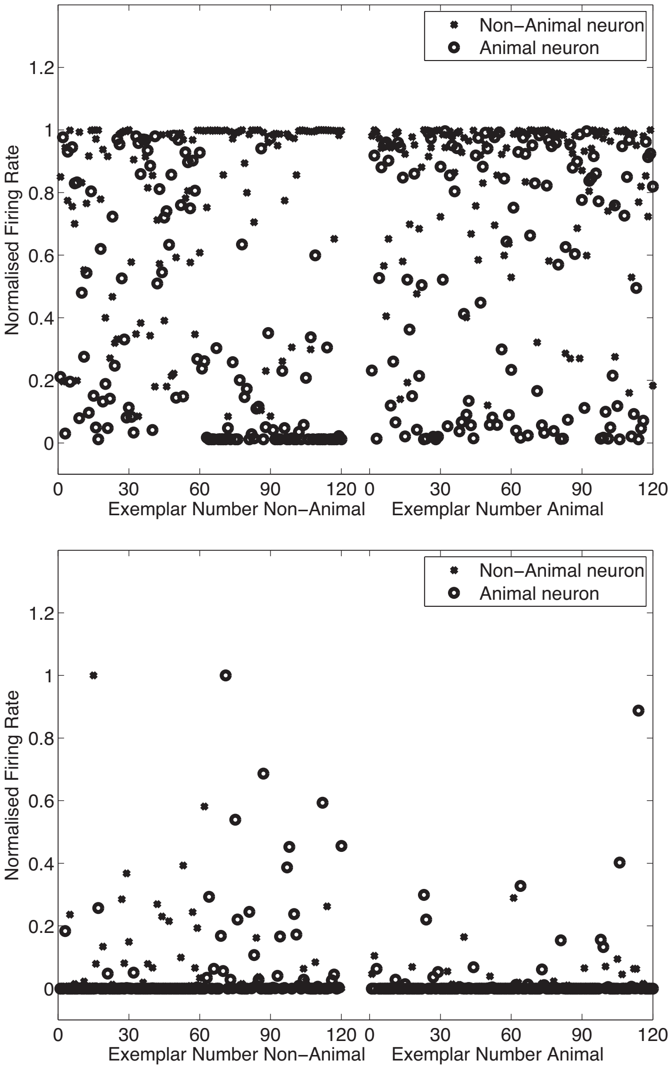

Figure 2: Top: Firing rate of two C3 units of HMAX in the Serre, Oliva and Poggio 2007 version when tested on the animals vs non-animals categorisation task. Bottom: Firing rate of two View Tuned Unit corresponding to S4 of HMAX when tested on the classes in the animals vs non-animals categorisation task. The neurons chosen were those with the highest single cell information that could be decoded from the responses of a neuron to 15 exemplars of each of the 2 classes (as well as a high firing rate) in the cross-validation design.
